# A chromosome-scale genome assembly of the tomato pathogen *Cladosporium fulvum* reveals a compartmentalized genome architecture and the presence of a dispensable chromosome

**DOI:** 10.1099/mgen.0.000819

**Published:** 2022-04-26

**Authors:** Alex Z. Zaccaron, Li-Hung Chen, Anastasios Samaras, Ioannis Stergiopoulos

**Affiliations:** ^1^​ Department of Plant Pathology, University of California Davis, Davis, USA; ^†^​ Present address: Department of Plant Pathology, National Chung Hsing University, Taichung, Taiwan, ROC

**Keywords:** Accessory chromosome, effectors, gene duplication, repeat-induced point mutation, fungal pathogen evolution, two-speed genome

## Abstract

*Cladosporium fulvum* is a fungal pathogen that causes leaf mould of tomato. The reference genome of this pathogen was released in 2012 but its high repetitive DNA content prevented a contiguous assembly and further prohibited the analysis of its genome architecture. In this study, we combined third generation sequencing technology with the Hi-C chromatin conformation capture technique, to produce a high-quality and near complete genome assembly and gene annotation of a Race 5 isolate of *C. fulvum*. The resulting genome assembly contained 67.17 Mb organized into 14 chromosomes (Chr1-to-Chr14), all of which were assembled telomere-to-telomere. The smallest of the chromosomes, Chr14, is only 460 kb in size and contains 25 genes that all encode hypothetical proteins. Notably, PCR assays revealed that Chr14 was absent in 19 out of 24 isolates of a world-wide collection of *C. fulvum*, indicating that Chr14 is dispensable. Thus, *C. fulvum* is currently the second species of Capnodiales shown to harbour dispensable chromosomes. The genome of *C. fulvum* Race 5 is 49.7 % repetitive and contains 14 690 predicted genes with an estimated completeness of 98.9%, currently one of the highest among the Capnodiales. Genome structure analysis revealed a compartmentalized architecture composed of gene-dense and repeat-poor regions interspersed with gene-sparse and repeat-rich regions. Nearly 39.2 % of the *C. fulvum* Race 5 genome is affected by Repeat-Induced Point (RIP) mutations and evidence of RIP leakage toward non-repetitive regions was observed in all chromosomes, indicating the RIP plays an important role in the evolution of this pathogen. Finally, 345 genes encoding candidate effectors were identified in *C. fulvum* Race 5, with a significant enrichment of their location in gene-sparse regions, in accordance with the ‘two-speed genome’ model of evolution. Overall, the new reference genome of *C. fulvum* presents several notable features and is a valuable resource for studies in plant pathogens.

## Data Summary

**Impact Statement**

Studies on the genome architecture of fungal pathogens can reveal large-scale architectural changes that greatly affect many aspects of microbial life in the context of adaptation and virulence. However, such studies are hampered by genomes containing large amounts of repetitive DNA, which prevents contiguous genome assemblies. In this study, we combined long-read sequencing with a chromatin conformation capture (3C)-based method to obtain a high-quality and near complete genome assembly of the fungal pathogen *Cladosporium fulvum* that causes tomato leaf mould. Comparative analysis with other fungal plant pathogens revealed an intriguing genome architecture characterized by genomic regions rich in genes that are interpolated with long intergenic regions rich in repetitive DNA but almost void of genes. Genes likely involved in pathogenicity are preferentially located in gene-sparse regions, which may provide a fast-evolving territory for their adaptive diversification. One of the assembled chromosomes was absent in several isolates from a world-wide collection of the fungus, indicating that the genome of *C. fulvum* is composed of core and dispensable chromosomes. Taken together, the results indicate that some fungal pathogens may have evolved a dynamic genome architecture that may provide advantages for their adaptation to adverse conditions.

This whole-genome project has been deposited at NCBI BioProject under the accession PRJNA565804. PacBio reads have been deposited at the NCBI Sequence Read Archive (SRA) under the accession SRR16292145. Illumina whole-genome sequencing, Hi-C, and RNA-seq reads have been deposited at the NCBI SRA under the accessions SRR16292144, SRR16292147, and SRR16292146, respectively. Pooled whole-genome sequencing of three isolates containing Chr14 and three isolates absent of Chr14 were deposited at the NCBI SRA under the accessions SRR18210015 and SRR18210014, respectively. The *C. fulvum* Race 5 chromosomes have been deposited at DDBJ/ENA/GenBank under the accessions CP090163 through CP090176. Scripts and code snippets utilized in this study were deposited in a public GitHub repository available at https://github.com/alexzaccaron/2021_cfr5_gm. Custom repetitive DNA libraries are available at https://doi.org/10.5281/zenodo.6380765.

## Introduction

Rapid advances in whole genome sequencing technologies over the last two decades have enabled the sequencing and comparative genome analysis of a wide range of fungal plant pathogens [1]. However, the majority of fungal genomes sequenced so far have mostly utilized short-read sequencing technologies which, while they excel at characterizing small DNA polymorphisms in populations, they have limited power when it comes to analysing repetitive genomic regions, phasing alleles, and inferring transposable element (TE) mobilization patterns and insertion sites [2, 3]. Such limitations, however, can significantly impede the study of higher-order genomic architectural features, such as segmental sequence duplications, translocations, copy number variations, and other chromosomal variations that can potentially affect several aspects of microbial life, including fitness, virulence, and adaptation to adverse environmental conditions [4–8]. These shortcomings can be overcome by third generation long-read sequencing and chromosome conformation capture (3C)-based techniques, which enable the *de novo* assembly of genomes to often chromosome level, thus allowing in-depth studies of genome architectures.

The increasing number of fungal genomes assembled to near-chromosome level has already revealed large differences in their architecture and organization, often even among genomes of phylogenetically closely-related fungal species or strains within a species [1, 9–11]. One of the most prominent features of the dynamic nature of fungal genomes is the presence of dispensable chromosomes in some species. Also known as accessory or B chromosomes, these chromosomes show a non-Mendelian mode of inheritance and they are present in some but not all individuals of a population [12]. In fungi, accessory chromosomes are typically small (<2 Mb), lack homology to chromosomes of phylogenetically closely-related species, are rich in repetitive DNA, and harbour a small number of genes that may or may not increase the fitness of the organism [13]. For instance, eight dispensable chromosomes have been described in the wheat pathogen *Zymoseptoria tritici* but a clear effect on fitness of the fungus has yet to be demonstrated for most of these chromosomes [6, 14]. In contrast, accessory chromosomes in *Fusarium* spp. and *Alternaria* spp. are enriched in effector genes and genes for the biosynthesis of host-selective toxins that can be transferred horizontally [15–19]. For example, *F. oxysporum* f. sp. *radicis-cucumerinum* has an accessory chromosome (chr^RC^) rich in repetitive DNA and candidate effector genes, one of which (*SIX6*) contributes to virulence toward cucumber [20]. Moreover, a non-pathogenic strain of *F. oxysporum* f. sp. *radicis-cucumerinum* turned pathogenic toward cucumber by acquiring chr^RC^ through horizontal chromosome transfer [20]. Thus, accessory chromosomes can potentially allow rapid adaptation of fungal pathogens to hosts.

Next to accessory chromosomes, various other structural variations have been observed in fungal genomes. For instance, species of Dothideomycetes, the largest class of the fungal kingdom that includes many economically important plant pathogens [21], show a particularly intriguing phenomenon of mesosynteny, which refers to the conservation of the gene content between species but in a randomized order and orientation on homologous chromosomes, presumably caused by extensive intrachromosomal and rare interchromosomal rearrangements [22, 23]. Although genes in fungal genomes can undergo extensive reshuffling via intrachromosomal rearrangements, genome compartmentalization has been observed as well, particularly with reference to virulence and pathogenicity-related genes, such as those encoding effector proteins [11, 24–26]. This is the case for example with smut fungi [27, 28], *Colletotrichum* spp. [29, 30], *Verticillium* spp. [31], and *Leptosphaeria* spp. [32, 33], among others [8, 34], in which effectors genes are found clustered within their genomes. Such clusters are often embedded in subtelomeric parts of the chromosomes, and other dynamic and fast-evolving regions of the genome that are generally characterized by a low gene density, low GC-content, and high abundance of repetitive DNA and TEs [8, 34]. This compartmentalized genome architecture characterized by repeat-rich regions enriched with candidate effector genes, and repeat-poor regions harbouring mostly housekeeping genes, has given rise to the so-called ‘two-speed genome’ model, which is thought to facilitate the rapid evolution and adaptive diversification of genes co-localizing in repeat-rich regions [24, 25, 35]. TEs have a profound impact on the evolution and the genomic architecture of such regions, as their activity promotes genetic variation and phenotypic diversity [4, 7, 36–38]. For example, gain-of-virulence was observed in a strain of the rice pathogen *Magnaporthe oryzae* that carried a nonfunctional copy of the effector gene *AvrPi9*, which was disrupted by the insertion of a TE in its coding sequence [39]. Horizontal transfer of the virulence gene *ToxA* mediated by TEs surrounding this gene has been reported among the wheat fungal pathogens *Parastagonospora nodorum*, *Pyrenophora tritici-repentis*, and *Bipolaris sorokiniana* [40]. Repetitive DNA has also been shown to accelerate genome evolution, particularly of effector genes, by the spillage of Repeat-Induced Point (RIP) mutations [33]. RIP is a premeiotic defence mechanism specific to fungal genomes that hypermutates repetitive DNA by inducing C-to-T changes during sexual reproduction [41–43]. Effector genes in *Leptosphaeria maculans* were shown to carry RIP mutations that ‘leaked’ from repeats in close physical proximity [33]. These examples indicate that a genome architecture characterized by the co-localization of genes important for pathogenicity or host adaptation and repetitive DNA, potentially enhances pathogen virulence and adaptation to hosts [11, 24, 35].

*Cladosporium fulvum* (syn. *Passalora fulva*, syn. *Fulvia fulva*) is a non-obligate biotrophic fungal pathogen (Ascomycetes; Dothideomycetes; Capnodiales) and the causal agent of the tomato leaf mould [44]. Although the disease is nowadays mostly of local interest to some parts of the world, the pathogen has been extensively used as a model species to study plant-microbe interactions [45, 46] and is the first fungus from which an avirulence (*Avr*) gene was ever cloned [47]. To date at least 12 effectors, i.e. Avr2, Avr4, Avr4E, Avr5, Avr9, Ecp1, Ecp2, Ecp2-2, Ecp2-3, Ecp4, Ecp5, and Ecp6 have been cloned from this pathogen and are shown to be avirulence determinants in tomato accessions with matching *Cf* resistance genes [48], although many more effector encoding genes have been identified *in silico* in its genome [49]. The first and so far only genome of *C. fulvum* was assembled and annotated nearly a decade ago [49] and although the genome of *C. fulvum* isolate 0WU has provided ample insights into the biology of the fungus, its high repetitive DNA content prohibited a contiguous assembly based on short-read sequencing. Consequently, the resulting highly fragmented assembly [49] prevented the study of the genome architecture of *C. fulvum* and the mapping of its genes to chromosomes.

In this study, we combined the PacBio single-molecule real-time (SMRT) sequencing technology [50] with Hi-C chromatin conformation capture [51] to obtain a high-quality and nearly complete genome assembly for *C. fulvum* isolate Race 5 Kim (hereafter *C. fulvum* Race 5) [52]. The resulting assembly contains 14 chromosomes (Chr1-to-Chr14), ten of which have been assembled telomere-to-telomere. Genomic analyses revealed a compartmentalized genome architecture composed of gene-dense regions interspersed with repeat-rich regions. PCR assays revealed that Chr14, the smallest of the chromosomes in *C. fulvum*, is present in a few but missing in several *C. fulvum* isolates, indicating that this chromosome is dispensable. The new reference genome of *C. fulvum* presented herein is a considerable improvement over the previous reference genome of isolate 0WU [49] and is a valuable resource for future functional and comparative genomic studies.

## Methods

### Fungal isolates, nucleic acid extractions, and sequencing

*Cladosporium fulvum* Race 5 Kim, an isolate of the fungus that was initially isolated in France in 1979 [52], was kindly provided by Emeritus Professor Pierre J. G. M. De Wit, Laboratory of Phytopathology at Wageningen University in the Netherlands. An additional twenty-four isolates of *C. fulvum* [52], were kindly provided by Professor Matthieu H. A. J. Joosten from the same laboratory.

High-molecular weight (HMW) genomic DNA from *C. fulvum* Race 5 was isolated according to Jones *et al*. 2019 [53] with some modifications. Specifically, *C. fulvum* Race 5 was grown on potato dextrose agar (PDA), at 22 °C for 2 weeks. Spores were harvested from the PDA plates, and 10^6^ spores were inoculated in 100 ml Gamborg’s B5 medium and grown at 22 °C for 1 week. The mycelia were filtered by two layers of cheesecloth and freeze-dried for 2 days. The dried material was ground with mortar and pestle in liquid nitrogen. An amount of 500 mg of starting material was then mixed with 17.5 ml of the lysis buffer, containing 10 kU of RNase A (ThermoFisher Scientific; Catalogue #: EN0531), 6.5 ml of buffer A (0.35 M sorbitol in 0.1 M Tris-HCl, pH 9, and 5 mM EDTA, pH 8), 6.5 ml of buffer B (0.2 M Tris-HCl, pH 9, 50 mM EDTA, pH 8, 2 M NaCl, and 2 % CTAB), and 2.75 ml of buffer C (5 % N-lauroylsarcosine sodium salt). The sample was then incubated at 25 °C for 30 mins and inverted every 5 mins. A total of 200 µl of Proteinase K (New England BioLabs Inc.; Catalogue #: P8107S) was next added to the sample and incubated at 25 °C for 30 mins, while mixing by inversion every 5 min. After this step, 3.5 ml of 5 M potassium acetate was added to the sample, and the sample was incubated on ice for 5 mins. The sample was then spun at 5000 ***g***, at 4 °C, for 12 mins. The supernatant was transferred to a 50 ml tube containing 17.5 ml of phenol:chloroform:isoamyl alcohol (25 : 24 : 1, v/v, Sigma-Aldrich, Catalogue #: P3803) and mixed by inversion for 2 mins. The sample was centrifuged at 4000 ***g***, at 4 °C, for 10 mins, and the phenol:chloroform:isoamyl alcohol separation was repeated again. The supernatant was mixed with 1.8 ml 3 M sodium acetate and 18 ml isopropanol, and then incubated at −20 °C overnight. The mixture was centrifuged at 10000 ***g***, 4 °C for 30 mins, and the pellet was transferred to a 1.7 ml tube. The pellet was centrifuged at 13000 ***g*** for 5 mins. After removing the supernatant, the pellet was washed with 70 % ethanol and then centrifuged at 13000 ***g*** for 5 mins. The ethanol wash step was repeated once, and the pellet was air-dried for 5 mins. Subsequently, the pellet was dissolved in 200 µl of 10 mM Tris, pH8.5. The DNA was further cleaned up by using AMpure XP beads (Beckman, Coulter Inc., Catalogue #: A63880) following the manufacturer’s instructions. The DNA was quantified using a Qubit dsDNA broad range (BR) assay kit (ThermoFisher Scientific, Catalogue #: Q32850) and its quality was measured with a Nanodrop ND-1000 (ThermoFisher Scientific) instrument based on the 260/280 and 260/230 ratios.

Library construction and sequencing of highly pure and HMW DNA was outsourced to the DNA Technologies and Expression Analysis Core Laboratory at the UC Davis Genome Centre (https://dnatech.genomecenter.ucdavis.edu/). The sample was enriched for HMW fragments prior to library construction by size selection of fragments longer than 20 kb, using the BluePippin pulsed-field gel electrophoresis platform (Sage Science, Beverly, MA). The constructed library was then sequenced using one SMRT Cell 1M v2 on a Sequel Chemistry v2 platform (Pacific Biosciences, Menlo Park, CA) with 10 h of total movie time. DNA extracted from *C. fulvum* Race 5 was also used to generate an Illumina library. In addition, approximately 300 mg of fresh weight of *C. fulvum* Race 5 grown as described above, was used for Hi-C library construction using the Proximo Hi-C Kit (microbial) (Phase Genomics), according to the manufacturer’s instructions. Illumina whole-genome sequencing, Hi-C, and RNA-seq libraries (see below) were sequenced on a NovaSeq 6000 instrument (PE150 format) utilizing 1.43, 2.35, and 9.78 % of a lane, respectively.

Total RNA from *C. fulvum* Race 5 strain was extracted using the Trizol Reagent (Invitrogen, Catalogue #: 15596026) according to the manufacturer’s instructions. Briefly, 10^6^ fungal spores were inoculated in 100 ml Gamborg’s B5 medium with vitamins at 200 r.p.m., at 22 °C for 6 days. The collected mycelia were subsequently inoculated in thirteen induction conditions for an additional 20 h. These induction conditions included growth in (i) 100 ml of rich medium (10 g l^−1^ yeast extract, 30 g l^−1^ glucose), (ii) 100 ml of minimal medium (1 g l^−1^ KH_2_PO_4_, 1 g l^−1^ KNO_3_, 0.5 g l^−1^ MgSO_4_.7H_2_O, 0.5 g l^−1^ KCl, 0.5 g l^−1^ sucrose, and 0.5 g l^−1^ glucose), (iii) 100 ml of Gamborg’s B5 medium with vitamins at 4 °C, or (iv) 42 °C for 4 days, 100 ml of Gamborg’s B5 medium supplemented with (v) 10 mg l^−1^ thiamine, (vi) 2 mg ml^−1^ sorbitol, (vii) 2 mg ml^−1^ maltose, (viii) 2 mg ml^−1^ xylose, (ix) 10 mM ammonia sulphate, (x) 5 mM H_2_O_2_, (xi) 5 mM methanol, (xii) 0.5M glutamine, and (xiii) 100 ml of Gamborg’s B5 medium without carbon source. The mycelia were collected by filtering through two layers of cheesecloth and ground into fine powders using a mortar and pestle, and liquid nitrogen. Then 100 mg of fine powder was mixed with 1 ml Trizol reagent by vortexing and RNA was extracted according to the manufacturer’s instructions. The RNA was quantified using a Qubit RNA broad range (BR) assay kit (ThermoFisher Scientific, Catalogue #: Q10210) and its quality was measured with a Nanodrop ND-1000 (ThermoFisher Scientific) instrument based on the 260/280 and 260/230 ratios. Finally, the RNA samples were pooled in equimolar amounts into a single sample prior to Illumina library construction, which was outsourced to the DNA Technologies and Expression Analysis Core of the UC Davis Genome Centre. Samples were sequenced (PE150 format) on a NovaSeq 6000 instrument as described above.

### Genome assembly

The genome of *C. fulvum* Race 5 was assembled with Canu v1.8 [54] with parameters *genomeSize=70* m, *corOutCoverage=60*, *minReadLength=5000*, *minOverlapLength=*3000, *corMinCoverage=5*, *corMhapSensitivity=normal*, and *correctedErrorRate=0.03*. Bacterial contigs were identified using the *sendsketch.sh* script from BBMap v38 [55], and the contig containing the mitochondrial genome was identified by querying the mitochondrial genome of *Z. tritici* [56] with BLASTn. Contigs were polished with Arrow v2.3.3 (https://github.com/PacificBiosciences/pbbioconda) based on PacBio reads mapped with pbmm2 v1.0.0 (https://github.com/PacificBiosciences/pbmm2). To further polish the contigs, Illumina reads were obtained and trimmed with fastp v0.20.1 [57]. Trimmed reads were mapped with BWA-MEM v0.7.17-r1188 [58], PCR duplicates were marked with samblaster v0.1.24 [59], and polishing was carried out with Pilon v1.23 [60]. Assembled contigs missing a telomere in one of their ends were extend up to 207 bp until the telomeric repeat was reached. To do so, Illumina reads mapping to the last or first 250 bp of contigs’ ends that were missing telomeres, were extracted with SAMtools and the script *filterbyname.sh* from BBMap v38. The extracted read pairs were then assembled with SPAdes v3.15.3 [61] with k-mer values of 33, 55, 77, and 111, and the assembled fragments, which contained telomeric repeats, were merged manually with the respective contigs. The Illumina reads were also used to estimate the genome size with the *kmercountexact.sh* script from BBMap v38 using a k-mer value of 31. To predict the chromosomes of *C. fulvum* Race 5, sequenced Hi-C reads were mapped to the assembled contigs with BWA-MEM v0.7.17-r1188 with parameters *−5, -S,* and *-P* to allow mapping of each read end individually. Mapped reads were processed with samblaster v0.1.24 [59] to mark PCR duplicates, and then with SAMtools v1.9 [62] to remove mapped reads with mate unmapped, not primary or supplementary alignments (SAM flag=2316). The scripts *makeAgpFromFasta.py* and *agp2assembly.py* (https://github.com/phasegenomics/juicebox_scripts) were then used to create an *assembly* file. Links were generated with matlock (https://github.com/phasegenomics/matlock) with parameter *bam2juicer*. A *hic* file was then produced from the *assembly* file and the links with the script *run-assembly-visualizer.sh* from the 3D-DNA package [63]. The Hi-C heat map was visualized and exported with Juicebox v1.11.08 [64].

### Genome assembly evaluation

To estimate the integrity of the assembly and possible misassemblies, the trimmed Illumina and raw PacBio reads were mapped to the assembly with BWA-MEM v0.7.17-r1188 with default parameters (for Illumina) and parameters *-M* and *-x pacbio* (for PacBio). PCR duplicates were marked with samblaster v0.1.24 [59]. The number of mapped reads and properly paired Illumina reads were determined with *flagstat* from SAMtools v1.9 [62]. Sniffles v1.0.12 [65] was used to predict structural variants based on mapped PacBio reads with default settings. PacBio and Illumina read coverage was examined with IGV v2.6.1 [65] at locations of predicted structural variations. Collapsed regions were identified based on the genome-wide coverage of PacBio and Illumina reads obtained with mosdepth v0.3.2 [66], using a sliding window of 30 kb.

### Repetitive DNA annotation

Custom *de novo* libraries of repetitive DNA were obtained with RepeatModeler v1.0.11 using the *ncbi* engine and RepeatModeler v2.0.2 with the parameter *-LTRStruct* enabled to run the LTR structural discovery pipeline [67]. The produced consensus repeats libraries were queried with InterProScan v5.32–71.0 to search for conserved domains not related to transposons that could have been called as repetitive DNA. Repeats were then masked with RepeatMasker v4.0.7 using the consensus libraries produced by RepeatModeler and with parameters adjusted for higher sensitivity (-s), to output alignments (*-a*), and repeat coordinates in GFF format (*-gff*). The custom repetitive DNA libraries and repeat coordinates are available at https://doi.org/10.5281/zenodo.6380765. The custom repeat libraries and the repeat alignments produced by RepeatMasker were used to estimate repeat divergence with the *parseRM.pl* script from the Parsing-RepeatMasker-Outputs package (https://github.com/4ureliek/Parsing-RepeatMasker-Outputs) with parameters *--land 50,1*, *--parse*, *--fa*, and *--nrem*. Genomic regions affected by RIP were identified with RIPper [68], using a 1 kb sliding window and a step size of 500 bp. Windows with substrate index value (CpA +TpG)/(ApC +GpT)≤0.75, product index value (TpA/ApT)≥1.1, and composite index value (TpA/ApT) – ([CpA +TpG]/[ApC +GpT])≥0.01 were considered to be affected by RIP [68]. The percentage of masked bases covered by RIPped windows was used to estimate the level of RIP mutations in repetitive regions. Windows considered as RIPped were queried with BLASTn against the genome using e-value <1E-20, identity >50 %, and query coverage >20 %. Based on these cutoff values, RIPped windows with a single BLASTn hit were considered single-copy, and therefore used as evidence of RIP leakage.

### Gene prediction

RNA-seq reads were processed with fastp v0.20.1 [57] to trim the adapters and low-quality sequences. Reads were then mapped to the *C. fulvum* Race 5 genome with HISAT2 v2.2.0 [69] using a maximum intron length of 3000 bp and the option *--dta* enabled to report alignments tailored for transcriptome reconstruction. Full length transcripts were then assembled with Stringtie v2.1.1 [70]. Genes were predicted with the Maker pipeline v2.31.10 [71]. Initially, the assembled transcripts of *C. fulvum* Race 5 and protein sequences from *Z. tritici* isolate IPO323 (GCF_000219625.1) and *Cercospora beticola* isolate 09–40 (GCF_002742065.1) were used by Maker to produce gene models in order to train the *ab initio* gene predictors Augustus v3.2.3 [72] and SNAP v2013-11-29 [73]. The script *maker2zff* that is incorporated in the SNAP software was used with parameters *-c 1 -o 1 x 0.1* to extract 3925 high-confidence gene models, which were then used to train again Augustus and SNAP. After parameter optimization, Augustus reported a sensitivity and a specificity at the nucleotide level of 0.968 and 0.836, respectively based on a testing data set that consisted of 200 genes. To further assist Maker predictions, gene models were also obtained with GeMoMa v1.6.3 [74]. GeMoMa mapped the gene annotations of *Z. tritici* IPO323 (GCF_000219625.1), *C. beticola* 09–40, and *C. fulvum* 0WU (JGI) with TBLASTn to the assembly of *C. fulvum* Race 5, and used the mapped RNA-seq reads to infer gene models with accurate exon-intron structure. The gene models produced with GeMoMa were filtered using GAF to keep only one isoform per gene (parameter *m=1*). The script *bam2hints* that is incorporated in the Braker software v2.1.5 [75] was used to extract intron hints from the mapped RNA-seq reads, using a minimum and a maximum intron length of 20 and 2000 bp, respectively. These intron hints and the gene models produced by GeMoMa using *C. beticola* as reference were used to train GeneMark v4.57 [76] with parameters *--fungus --training --soft_mask auto*. Finally, the pre-identified repeats and *gff* format along with all lines of gene evidence, i.e. assembled transcripts of *C. fulvum* Race 5, protein sequences from *Z. tritici* IPO323 (GCF_000219625.1) and *C. beticola* 09–40 (GCF_002742065.1), GeMoMa gene models, trained predictors Augustus, SNAP, and GeneMark, were provided to Maker to select the best gene models for *C. fulvum* Race 5. Splice sites were extracted from mapped RNA-seq reads with RegTools v0.5.2 [77] with minimum intron size of 20 bp, maximum intron size of 3000 bp, and minimum anchor length of 8 bp. The splice sites were annotated with RegTools and genes with splice sites fully supported (i.e. known donor-acceptor [DA]) by at least five reads were used to estimate exon-intron prediction accuracy.

### Gene annotation

Gene annotation completeness was estimated with BUSCO (Benchmarking Universal Single-Copy Orthologs) v5.2.1 [78] using hmmsearch v3.1 and the database Dothideomycetes_db10 2020-08-05 as reference. Genes encoding key enzymes for secondary metabolism were identified with antiSMASH v6.0.1 [79]. Genes encoding carbohydrate-active enzymes (CAZymes) were identified and classified with dbCAN2 meta server [80], using the HMM database v9. Genes encoding proteases and transporters were identified based on homology searches performed with BLASTp (e-value <1E-10) against the MEROPS database v12 [81] and the transporter classification database (TCDB; 2021-06-20) [82], respectively. Proteases and transporters were classified based on the most homologous sequence according to BLASTp. Secreted proteins were identified with SignalP v5 [83] and transmembrane domains were identified with TMHMM v2 [84]. GPI-anchored proteins were identified with PredGPI [85], using PFrate <0.005 as threshold. Candidate effectors were characterized as secreted proteins and classified as effectors with EffectorP v2 [86]. We also considered as candidate effectors small secreted proteins shorter than 250 aa, with at least 2 % cysteine residues, no transmembrane domain in the mature protein, and no GPI anchor. Previously described candidate effectors from *C. fulvum* 0WU were obtained from NCBI and mapped to the genome of *C. fulvum* Race 5 with minimap2 v2.16 [87] in splice aware mode (parameter *-x splice*). Missing or inaccurate gene annotation of these candidate effectors in *C. fulvum* Race 5 were manually curated when necessary, based on mapped RNA-seq reads of *C. fulvum* Race 5. Differences in gene content among the chromosomes were analysed with a principal component analysis performed with the *prcomp* function (parameter *scale=TRUE*) and visualized with the *biplot* function within R v4.2.1.

### Compartmentalization analysis

The intergenic regions were obtained using the script *complement* from BEDtools v2.29.0 [88] to obtain genomic space not covered by genes. Subsequently, the script *closest* from BEDtools v2.29.0 was used to assign up- and downstream intergenic regions to each gene. Heat maps of the intergenic regions were obtained using the *geom_hex* function from the R package ggplot2 v3.3.3 [89] with a bin size of 50 within R v3.5.1. Clusters of genes based on intergenic sizes were obtained with the script *cluster* from BEDtools v2.29.0 by varying the maximum distance parameter (*-d*). Enrichment of PFAM domains of genes in gene-sparse regions was carried out with the *enricher* function from the R package clusterProfiler v3.18.1 [90], using the Benjamini and Hochberg *p*-value correction method and adjusted *p*-value<0.05. Enrichment of specific gene categories within gene-sparse regions was performed with the *phyper* function within R v4.0.3.

### Comparative analyses with other genomes

The genome of *C. fulvum* was aligned with the genome of other Dothideomycetes using PROmer from the MUMmer package v4.0 [91] with default settings. Alignments were then filtered with the *delta-filter* script that is incorporated in MUMmer to only retain the best matches (parameter *−1*). Alignment coordinates were used to make dot plots within R v3.5.1 and a circos plot with circos v0.69–8 [92]. Assembled scaffolds of *C. fulvum* 0WU were split into contigs with the *splitasm* function from RagTag v2.0.1 [93]. The resulting contigs were mapped to the *C. fulvum* Race 5 assembly with minimap2 v2.20 [87] with parameters *-ax asm10*. The alignment was filtered with SAMtools v1.9 [61] to remove unmapped contigs and not primary alignment (SAM flag=260). Regions uncovered by the mapped contigs were determined with the *genomecov* function from BEDtools v2.29.0 [87]. The nucleotide sequences of genes from *C. fulvum* Race 5 were queried with BLASTn v2.12.0 with e-value <1E-5 and query coverage of at least 50 % against the scaffolds of *C. fulvum* 0WU. Genes with no BLASTn hit were considered missing in the genome of *C. fulvum* 0WU. To obtain evidence of expression of genes missing in the genome assembly of *C. fulvum* 0WU, RNA-seq reads of this same isolate were obtained from NCBI SRA database (SRR1171044, SRR1171045, and SRR1171046) and mapped to the genome of *C. fulvum* Race 5 with HISAT2 v2.2.1 [69] with parameter *--max-intronlen 3000*. Reads mapped to genes were counted with *featureCounts* from the Subread package v2.0.1 [94], and transcripts per million (TPM) values were then calculated with a custom R script available at http://github.com/alexzaccaron/2021_cfr5_gm/tree/main/gene_expression/scripts.

### Detection and confirmation of the mini-chromosome Chr14 in a population of *C. fulvum*

Four pairs of primers were designed to PCR-amplify eight predicted genes in four different regions of Chr14 (Table S1 and Fig. S1a, available in the online version of this article). Isolates were grown in PDA media for 10 days at 25 °C. Spores and mycelial fragments were harvested from the media surface using sterile blades. DNA was extracted using a simple SDS based extraction [95]. The PCR reactions were performed using an Apex Red Mix (APEX Bioresearch Products, USA) following the manufacturer instructions. Cycling conditions consisted of 35 cycles of 30 s at 94 °C, 30 s at 55 °C, 56 °C, or 57 °C depending on the primer combination, and 2 min at 72 °C. A final 7 min extension step at 72 °C completed the reaction. PCR products were visualized in a 1 % agarose gel (Fig. S1b). To further confirm dispensability of chromosome Chr14, DNA from isolates for which Chr14 was predicted to be present (isolates 2, IMI Argent 358 077, and Turk 1 a) or absent (isolates IPO 2.4.8.9.11 Polen, IPO 249 France, and 2.5) was pooled in equimolar amounts into two samples. DNA libraries were prepared using the Invitrogen Collibri ES DNA Library Prep Kit for Illumina Systems (Thermo Fisher Scientific) according to the manufacturer’s instructions (protocol MAN001845). Libraries were multiplexed using unique dual indexes and sequenced at the UC Davis Genome Centre on an Illumina NovaSeq 6000 instrument (PE150 format). Reads were trimmed with fastp v0.23.1 [57] and mapped to the genome assembly of *C. fulvum* Race 5 with BWA-MEM v0.7.17 [58]. Read depth across chromosome Chr14 was determined with *mosdepth* v0.3.3 [66].

## Results

### A chromosome-level and nearly complete genome assembly of *C. fulvum* Race 5

To produce a high-quality genome assembly for *C. fulvum* Race 5, a workflow was employed that combined PacBio reads, Illumina reads, and Hi-C chromatin conformation capture (Fig. S2). Initially, the genome of *C. fulvum* Race 5 was sequenced on a PacBio Sequel I platform, which produced a total of 583 199 reads (8.5 Gbp of data) with an average read length of 14638 bp (~125 × coverage). The assembler Canu [54] assembled the PacBio reads into 43 contigs, with a total length of 80.5 Mb. Of these, 29 contigs were removed from the assembly because they matched to bacterial genomes (24 contigs with a combined size of 12.9 Mb), were contained within other contigs, or were formed from a single PacBio read (four contigs with a combined size of 186.4 kb) (Table S2), or corresponded to the mitochondrial genome of *C. fulvum* (one contig of 179.1 kb in size). To further improve the quality of the assembly, two rounds of polishing were carried out on Pilon with 97.4M Illumina reads that were obtained, which performed 692 changes in the first round and four changes in the second round. The resulting assembly of *C. fulvum* Race 5 contained 14 contigs totaling 67.17 Mb in size, with an L_50_ of 5 and an N_50_ of 5.7 Mb. Notably, these assembly contiguity metrics are a considerable improvement over the previous reference genome of *C. fulvum* isolate 0WU (Table 1). Further genome size estimation based on k-mer counting of contamination-free Illumina reads indicated a genome size of 66.53 Mb, which is in agreement with the size of the obtained assembly. To verify the integrity of the assembly and identify potential misassemblies, the Illumina reads were trimmed and mapped to the polished contigs. Mapping was realized at an alignment rate of 99.67%, with 99.3 % of the reads properly paired and 90.21 % uniquely mapped. Of these, 91.76 % mapped to the nuclear chromosomes and 7.9 % mapped to the mitochondrial contig. Next, the PacBio reads were mapped to the assembled contigs and structural variants (SVs) were called with Sniffles [65], which identified only two possible SVs in the nuclear contigs. However, these SVs were not supported by the Illumina reads (Fig. S3). Moreover, analysis of the Illumina and PacBio coverage revealed only three possible collapsed regions located in three different contigs, with one such collapsed region co-localizing with the 18 S-5.8S-28S rDNA locus (Fig. S4). Collectively, these results indicate that the obtained assembly of *C. fulvum* Race 5 is essentially free of major misassembly errors. As a final verification step, the obtained Hi-C data were used to evaluate the correctness of the assembly. A total of 80M Hi-C reads were produced and aligned to the genome of *C. fulvum* Race 5 with an alignment rate of 98.4 %. Interaction intensity by proximity ligation supported that the 14 contigs were distinct chromosomes of *C. fulvum* Race 5 with no visible misassemblies (Fig. S5a). Thus, the 14 assembled contigs represent individual chromosomes (Chr) and henceforth will be referred to as Chr1-to-Chr14, according to their size, from largest to smallest.

**Table 1.** Genome assembly statistics of *Cladosporium fulvum* Race 5 compared to the previous reference genome assembly of *C. fulvum* isolate 0WU

Assembly statistics	*C. fulvum* Race 5	*C. fulvum* 0WU
Assembly size (bp)	67 169 167	61 113 266
Scaffolds	14	4865
Contigs	14	5715
Scaffold N_50_	5 777 465	56 512
Scaffold L_50_	5	250
Scaffold N_90_	3 311 397	5928
Scaffold L_90_	11	1756
Longest scaffold	11 362 290	530 628
GC (%)	48.94	48.78
Number of gaps	0	850
Gapped bases	0	320 683

### Overall properties of the *C. fulvum* Race 5 chromosomes

**Fig. 1.**
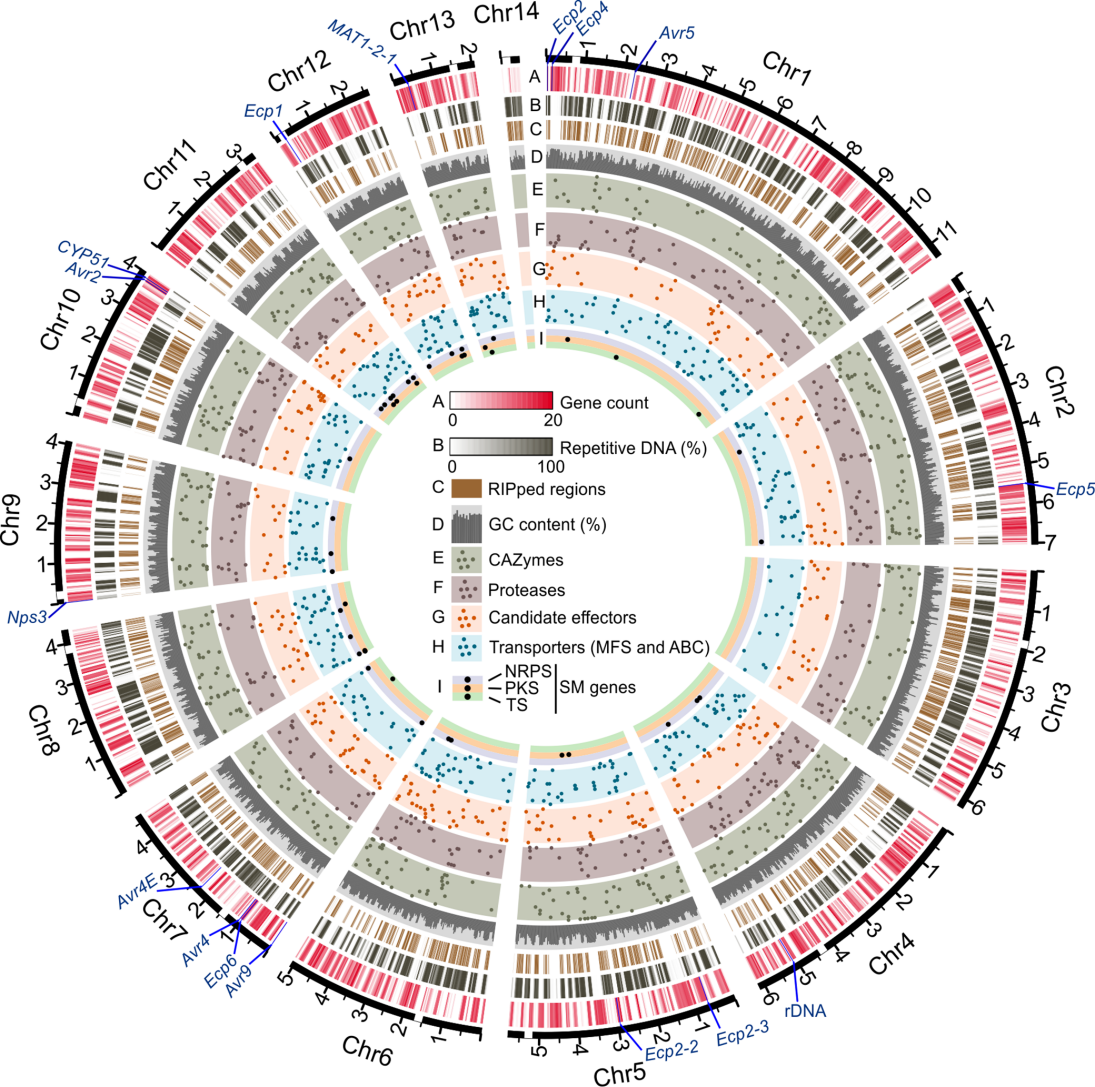
Chromosomes of *Cladosporium fulvum* Race 5. The circos plot shows the assembled chromosomes (solid black lines) with tracks representing (**a**) protein-coding genes, (**b**) repetitive DNA content, (**c**) regions affected by Repeat-Induced Point (RIP) mutations, (**d**) GC content from 30–60 %, (**e**) the location of genes encoding carbohydrate-active enzymes (CAZymes), (**f**) the location of genes encoding proteases, (**g**) the location of genes encoding candidate effectors, (**h**) the location of genes encoding transporters from the major facilitator superfamily (MFS) and ATP-binding cassette (ABC) family, and (**i**) the location of genes encoding key enzymes for secondary metabolism, i.e. non-ribosomal peptide synthetases (NRPS), polyketide synthases (PKS), and terpene synthases (TS). Gene locations are represented by points, and points in tracks (**e, f, g, h**) were randomly distributed on the perpendicular axis. The location of a few genes of general interest is indicated in the outermost track. These genes include previously described avirulence (*Avr*) and extracellular protein encoding genes (*Ecp*), the 18 S-5.8S-28S rDNA, the mating type 2 (MAT1-2) locus, and *CYP51* that encodes the target enzyme of demethylation inhibitor (DMI) fungicides. The approximate location of each centromere is indicated with a white rectangle on the outermost axis and major tick marks represent Mb. Gene count (**a**), repetitive DNA (**b**), and GC content (**d**) were determined using a sliding window of 30 kb. The figure shows that the chromosomes of *C. fulvum* Race five are composed of gene-rich and repeat-poor regions that are interspersed with gene-poor and repeat-rich regions, in accordance with the ‘two-speed genome’ model. The figure also shows that a large portion of the genome of *C. fulvum* Race 5 is affected by RIP mutations and that genes involved in secondary metabolism are preferentially located in smaller chromosomes.

The 14 assembled chromosomes of *C. fulvum* Race 5 vary in size from 0.46 Mb to 11.36 Mb (Fig. 1 and Table 2). Notably, at 11.36 Mb, Chr1 is considerably larger in size than the other chromosomes, which are at most 7.0 Mb long. All assembled chromosomes have the canonical telomeric repeat 5′-TTAGGG-3′ at both ends, indicating that they were assembled end-to-end (Fig. S5b and Table 2). As previously reported in other fungi, the Hi-C interaction frequency indicated the approximate location of centromeres [96–99]. In *C. fulvum*, centromeres are putatively located proximal to chromosome ends (Fig. S5b). Specifically, Chr1, Chr2, Chr5, Chr8, Chr9, Chr10, Chr11, and Chr12 are acrocentric, whereas Chr3, Chr4, Chr6, Chr7, Chr13, and Chr14 are submetacentric [100]. The distribution of protein-coding genes and of repetitive DNA throughout the genome (described in detail in later subsections) revealed an idiosyncratic compartmentalized pattern of gene-rich, repeat-poor, and high GC regions that are interspersed by gene-poor, repeat-rich, and low GC regions (Fig. 1). However, Chr14 is an exception; at 460 kb, this chromosome is composed of nearly 80 % repeats and harbours only 25 predicted genes of unknown function. Such characteristics are typical of dispensable chromosomes in fungi [13], suggesting that Chr14 could be dispensable.

**Table 2.** Statistics of the chromosomes of *Cladosporium fulvum* Race 5. Copy number of telomeric repeats at the chromosomes’ immediate ends are indicated when present

Chromosome	Size (bp)	GC (%)	Genes	Gene density (genes/Mb)	Repeats (%)	Left telomere	Right telomere
Chr1	11 362 290	48.85	2322	204.4	52.5	(CCCTAA)x9	(TTAGGG)x7
Chr2	7 036 032	49.18	1577	224.1	48.6	(CCCTAA)x19	(TTAGGG)x7
Chr3	6 232 865	47.22	1040	166.9	61.6	(CCCTAA)x8	(TTAGGG)x21
Chr4	6 141 308	49.91	1491	242.8	44.6	(CCCTAA)x15	(TTAGGG)x7
Chr5	5 777 465	49.22	1237	214.1	49.8	(CCCTAA)x12	(TTAGGG)x9
Chr6	5 061 772	48.88	1137	224.6	50.2	(CCCTAA)x7	(TTAGGG)x7
Chr7	4 686 795	48.33	951	202.9	54.2	(CCCTAA)x6	(TTAGGG)x12
Chr8	4 340 606	48.48	913	210.3	53.9	(CCCTAA)x9	(TTAGGG)x7
Chr9	4 070 492	49.83	1027	252.3	42.4	(CCCTAA)x8	(TTAGGG)x13
Chr10	4 017 737	48.77	851	211.8	49.3	(CCCTAA)x7	(TTAGGG)x15
Chr11	3 311 397	49.6	850	256.7	40.9	(CCCTAA)x32	(TTAGGG)x10
Chr12	2 606 583	50.21	698	267.8	38.3	(CCCTAA)x8	(TTAGGG)x5
Chr13	2 063 147	50.12	571	276.8	36.0	(CCCTAA)x12	(TTAGGG)x11
Chr14	460 486	45.63	25	54.3	77.9	(CCCTAA)x26	(TTAGGG)x5

### Repetitive regions in the genome of *C. fulvum* Race 5 are heavily affected by RIP

**Fig. 2.**
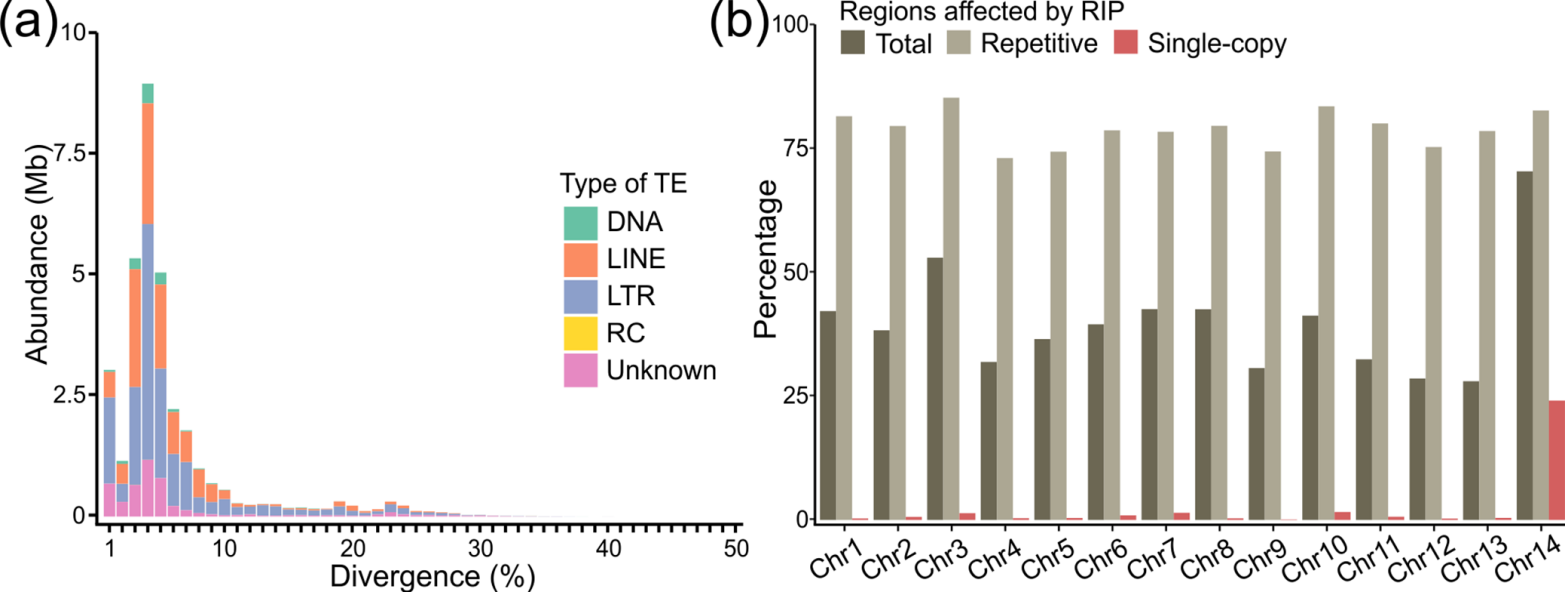
The repetitive DNA landscape of *Cladosporium fulvum* Race 5. (**a**) Bar plot showing the number of bases covered by predicted transposable elements (TEs) of different (sub)classes, i.e. DNA transposons (DNA), long interspersed nuclear elements (LINE), long terminal repeats (LTR), rolling-circles (RC), and unclassified TEs. The x-axis shows the divergence of repeats from the consensus sequences. The figure shows that the genome of *C. fulvum* Race 5 is abundant in repeats with an overall low divergence. (**b**) Bar plot showing the percentage of regions in the chromosomes of *C. fulvum* Race 5 that are predicted to be affected by Repeat-Induced Point (RIP) mutations. The figure shows that RIP affects approximately 40 % of all chromosomes and that RIP predominates in repeat-rich regions. However, single-copy regions are also predicted to be affected by RIP and particularly in the mini-chromosome 14 (Chr14).

*De novo* annotations of the repeats present in the genome of *C. fulvum* Race 5 with RepeatModeler v1.0.11 revealed that nearly half of the genome (33.4 Mb; 49.7%) is composed of interspersed repetitive DNA, in agreement with the estimated repeat content of 47.2 % for isolate 0WU [49]. Most repeats can be classified as retrotransposons (27.5 Mb; 40.9 % of the genome), and are more abundant than DNA transposons (1.2 Mb; 1.8 % of the genome) and unclassified repeats (4.7 Mb; 7.0 % of the genome) (Table S3). Moreover, 82 % of the regions putatively covered by transposable elements are composed of three abundant families, i.e. the LINE Tad1 family (16.3 % of the genome), the LTR Gypsy family (15.4 % of the genome), and the LTR Copia family (9.2 % of the genome). Comparative analysis of the repeats with their corresponding consensus sequences revealed that almost all repetitive sequences (29.8 Mb; 44.4 % of the genome) have an overall low nucleotide sequence divergence of less than 10 % (Fig. 2a). Indeed, most repeats exhibit a sequence divergence between 3 and 5 %, whereas a considerable amount of the genome (3 Mb; 4.5%) is covered by repeats with very low divergence of less than 1%, suggesting that these regions likely correspond to recently proliferated repeat families. To confirm these results, a similar analysis was conducted using the newer version of RepeatModeler v2.0.2 [67]. Indeed, both RepeatModeler v1 and RepeatModeler v2 predicted similar amounts of repetitive DNA, 49.7 and 49.8 %, respectively (Table S3). Moreover, in accordance with the findings obtained with RepeatModeler v1, analysis of the repeats identified with RepeatModeler v2 indicated that almost all repeats have divergence of less than 10%, and most repeats have divergence between 3 and 5 % (Fig. S6).

A sliding window analysis further revealed that 39.2 % of the genome of *C. fulvum* Race 5 is affected by RIP mutations (i.e. RIPed) [41, 43], in agreement with the previous estimate of 42.4 % for isolate 0WU [49]. In addition, 1532 Large RIP Affected Regions (LRARs) longer than 4 kb in size and with an average size of 16.7 kb could be detected. In comparison, a recent survey which analysed the occurrence of RIP in 58 fungal species indicated that all had less than 30 % of their genomes RIPed and contained at most 482 LRARs [101]. This indicates that *C. fulvum* is among the fungal species affected the most by RIP. However, depending on their repeat content, the chromosomes of *C. fulvum* Race 5 are RIPed at different extents, with Chr14 and Chr3 affected the most (70.4 and 53.0 %, respectively), and Chr12 and Chr13 affected the least (28.0 and 28.6 %, respectively) (Table S4). These differences in RIP levels among the chromosomes can be attributed to their differences in repetitive DNA content, as a positive correlation existed among the two (correlation coefficient *R*=0.99; *p*-value=6.8E-11) (Fig. S7). When considering only repetitive regions, then between 73.1 and 85.3 % of the repeats are affected by RIP (Fig. 2b). Evidence of RIP leakage toward non-repetitive regions was also observed in all chromosomes. Specifically, from Chr1 to Chr13, between 2.5 kb and 32.7 kb of single-copy regions are RIPed, with RIP levels ranging from 0.1–1.6 % of their unmasked bases. However, in the mini-chromosome Chr14, 24.5 kb of single-copy regions show evidence of RIP, with RIP levels corresponding to a larger percentage (24.1%) of unmasked bases in this chromosome (Fig. 2b and Table S4). This indicates that Chr14 is heavily affected by RIP and that RIP leakage occurs more frequently in Chr14 than in the other 13 chromosomes.

### An accurate and fairly complete gene annotation of *C. fulvum* Race 5 assisted by pooled RNA-seq data

To assist gene predictions and to further obtain evidence of gene expression, RNA was isolated from *C. fulvum* Race 5 grown in different stress conditions and sequenced on an Illumina NovaSeq 6000 platform (PE150 format) at a high depth. The 326.2 M reads obtained by transcriptome sequencing (98.5 Gbp of data) mapped to the genome at an alignment rate of 97.1 %. A total of 12 822 transcripts were assembled, which were then used together with other gene-supporting evidence (see Methods) to predict 14 690 genes. Analysis of the completeness of gene space using BUSCO genes revealed that the genome assembly and annotation of *C. fulvum* Race 5 was 98.9 % complete, which is higher than the completeness (95.9%) of the *C. fulvum* isolate 0WU (Table 3). Notably, compared to other 39 annotated genomes of Capnodiales available at NCBI, only *Z. tritici* isolate ST99_3D1 has currently a higher completeness (99.1%) than *C. fulvum* Race 5 at the protein level (Fig. S8). The average size of the 14 690 predicted genes in the genome of *C. fulvum* Race 5 is 1375 bp, with 5474 (37.3 %) genes being single-exon and 9216 (62.7 %) genes being multi-exon. Of the later ones, 7843 (85.1 %) genes have at least one splice-site supported by five or more RNA-seq reads, and 6935 (75.2 %) genes have all splice-sites supported by five or more RNA-seq reads, indicating accurate exon-intron structure prediction.

**Table 3.** Gene annotation statistics of *Cladosporium fulvum* Race 5 compared to the previous reference gene annotation of *C. fulvum* isolate 0WU

Gene annotation statistics	*C. fulvum* Race 5	*C. fulvum* 0WU
Number of genes	14 690	14 127
Number of single-exon genes	5474	5411
Number of multi-exon genes	9216	8716
Mean exons per gene	2.1	2.2
Total gene length (bp)	20 211 715	19 998 515
Total exon length (bp)	18 870 323	18 300 722
Total intron length (bp)	1 358 240	1 714 406
Mean gene length (bp)	1375	1415
Mean cds length (bp)	1284	1295
Mean exon length (bp)	598	595
Mean intron length (bp)	80	103
Complete BUSCOs (%)	98.9	95.9
Complete single-copy BUSCOs (%)	98.8	95.6
Complete duplicated BUSCOs (%)	0.1	0.3
Fragmented BUSCOs (%)	0.3	0.7
Missing BUSCOs (%)	0.8	3.4

Functional annotations with InterProScan indicated that of the 14 690 genes predicted in the genome of *C. fulvum* Race 5, 6316 (43.0 %) genes encode proteins with a conserved Pfam domain in their primary sequence. Homology searches performed with eggNOG (evolutionary genealogy of genes: non-supervised orthologous groups) further assigned 8884 (60.5 %) genes to a KOG (eukaryotic orthologous groups) category and 1686 (11.4 %) genes to a KEGG (Kyoto encyclopaedia of genes and genomes) Orthology (KO) ID group. Gene categories with relevance to fungal pathogens were also annotated. Specifically, 42 genes were predicted to encode key enzymes for the biosynthesis of secondary metabolites, including 11 polyketide synthases (PKSs), 15 non-ribosomal peptide synthetases (NRPSs), 11 NRPS-like fragments, one PKS-NRPS hybrid, and four terpene synthases (TSs) (Table S5). A total of 488 genes encoding CAZymes were identified as well, including 260 glycoside hydrolases (GHs), 106 glycosyltransferases (GTs), 30 carbohydrate esterases (CEs), 74 auxiliary activity enzymes (AAs), nine polysaccharide lyases (PLs), and nine CAZymes with single carbohydrate binding domains (CBMs) (Table S6). The genome of *C. fulvum* Race 5 further contains 359 proteases, divided into 177 serine, 83 metallo, 62 cysteine, 19 threonine, 14 aspartic, one asparagine, and three inhibitory peptidases (Table S7). A total of 2287 genes encoding putative transporters were also identified (Table S8), with the most abundant family of transporters being the major facilitator superfamily (MFS) (*n*=382), followed by the nuclear pore complex family (NPC) (*n*=121), the pore-forming NADPH-dependent 1-acyldihydroxyacetone phosphate reductase family (*n*=93), the equilibrative nucleoside (ENT) family (*n*=72), and the ATP-binding cassette (ABC) transporter family (*n*=57). Finally, the secretome of *C. fulvum* Race 5 is predicted to consist of 1320 proteins, including 229 CAZymes, 77 proteases, and 345 candidate effectors (Table S9). When mining the genome for candidate effector genes, it was observed that several genes (*n*=35) previously described as candidate effectors in *C. fulvum* [102] were absent in the gene annotation of *C. fulvum* Race 5. Most of these genes (*n*=31) were also absent in the reference annotation of *C. fulvum* 0WU, indicating that they are difficult to annotate based solely on *ab initio* predictions. Therefore, these genes were obtained from NCBI and manually annotated in the genome of *C. fulvum* Race 5. Lastly, another 69 previously described effector (e.g. *Avr2, Avr4, Avr9*) or candidate effector genes (e.g. *Ecp7, Ecp8, Ecp9-1*) in *C. fulvum* had their annotation verified and manually adjusted when needed based on mapped RNA-seq reads.

### Distribution of genes within the chromosomes of *C. fulvum* Race 5

**Fig. 3.**
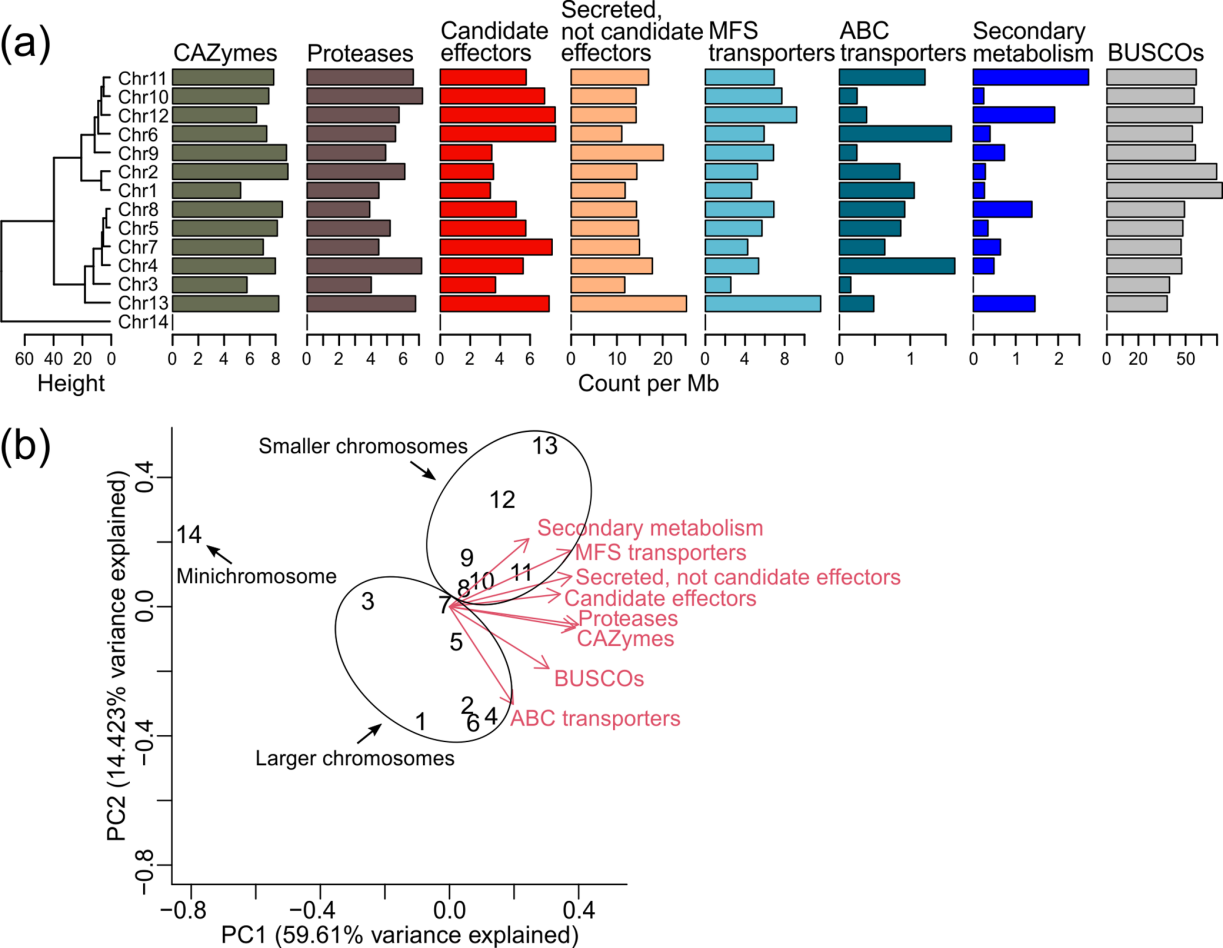
Large and small chromosomes of *Cladosporium fulvum* Race 5 differ in gene content. (**a**) Overall gene density, i.e. counts per million base pairs, of specific categories of genes in each chromosome. Chromosome were grouped using a hierarchical clustering performed using the complete method based on the Euclidean distances. (**b**) Principal component analysis biplot grouping the chromosomes based on gene density. The mini-chromosome 14 appears far from the others, as this chromosome contains only hypothetical genes. Chromosomes 1 to 13 can be organized into two groups based on their size. One group contains chromosome 1 to chromosome 7 (the seven largest chromosomes) which, overall, have higher densities of genes encoding ATP-binding cassette (ABC) transporters and BUSCO genes. Chromosome 8 to chromosome 13 have, overall, higher densities of genes encoding key enzymes for secondary metabolism and major facilitator superfamily (MFS) transporters.

The 14 chromosomes assembled in *C. fulvum* Race 5 vary in gene content (Table 2), with the mini-chromosome Chr14 being markedly different from the other 13 chromosomes in that it harbours only hypothetical genes. Notably, smaller chromosomes also have higher density of key genes involved in secondary metabolism (i.e. PKSs, NRPSs, and TSs) and genes encoding MFS transporters, whereas larger chromosomes have higher densities of genes encoding ABC transporters and BUSCO genes (Fig. 3a and b and Fig. S9). Genes encoding CAZymes, proteases, secreted proteins, and candidate effectors are not preferentially located in smaller or larger chromosomes (Fig. 3b).

When considering the distribution of genes on the chromosomes then, contrary to expectations, there was no substantial reduction in gene content in subtelomeric regions. Specifically, 143 genes were identified within 25 kb of the telomeric repeats (Table S10), representing a density of 204.3 genes per Mb, which is slightly below the gene density for the entire genome (218.7 genes per Mb). Interestingly, most of these genes (*n*=90) encode hypothetical proteins. Conversely, BUSCO genes are underrepresented in subtelomeric regions (*p*-value=1.7E-5), whereas the density in these regions of genes encoding proteases and key enzymes for secondary metabolism is similar to that of the whole genome (Table S11). Notably, one NRPS gene, previously described as *Nps3* [103], is the leftmost gene in Chr9, located at a distance of 15648 bp from the telomere (Fig. 1). In contrast to the gene categories described above, candidate effector genes are significantly enriched in subtelomeric regions (*p*-value=0.002) and exhibit a higher density (16.7 genes per Mb) in these regions, compared to the whole genome (5.1 genes per Mb) (Table S11). Among the effector genes present in subtelomeric regions is the avirulence gene *Avr9,* which is located 6545 bp upstream of the left-hand side telomere in Chr7, and nine other genes that encode the candidate effectors Ecp13, Ecp25, Ecp37, Ecp47, CE10, and CE29. Finally, another gene that is considerably close to a telomere, i.e. 123.7 kb from the right-hand side of the telomere in Chr10, is *CYP51* that is involved in ergosterol biosynthesis and is the target of the demethylase inhibitor (DMI) fungicides (Fig. 1).

Two mating-type idiomorphs, designated MAT1-1 and MAT1-2, have been described in *C. fulvum* [52]. In accordance to previous reports [52], a search for mating-type genes in the genome of *C. fulvum* Race 5 identified a *MAT1-2-1* gene encoding a DNA-binding domain of the high-mobility group (HMG), but not the alpha-domain-encoding gene *MAT1-1-1*. The *MAT1-2-1* gene is located in Chr13, the second smallest chromosome, and is flanked by the hypothetical genes *ORF1-1-2* and *ORF1-2-2* [52]. In most Ascomycetes, *MAT* genes are typically flanked by the *Apn2* and *SLA2* genes, but in *C. fulvum* only *Apn2* is located proximal to *MAT1-2-1*, whereas *SLA2* is near the opposite end of Chr13 at a distance of 1.43 Mb from *MAT1-2-1* (Fig. S10).

By querying the rDNA from *Neurospora crassa* (FJ360521) with BLASTn, the 18 S-5.8S-28S rDNA of *C. fulvum* was identified in Chr4. Five 5579 bp identical copies of this gene, tandemly arranged in a 44.5 kb locus could be assembled but because this region is collapsed in the assembly, the rDNA copy number in the assembly is underestimated. Indeed, by mapping the Illumina reads to one of the rDNA copies and normalizing the coverage by the median coverage of all genes, then the estimated rDNA copy number in *C. fulvum* Race 5 is 42, which is comparable to the predicted number of copies in other Ascomycetes [104].

### The genome of *C. fulvum* Race 5 is compartmentalized into gene clusters flanked by repeat-rich intergenic regions

**Fig. 4.**
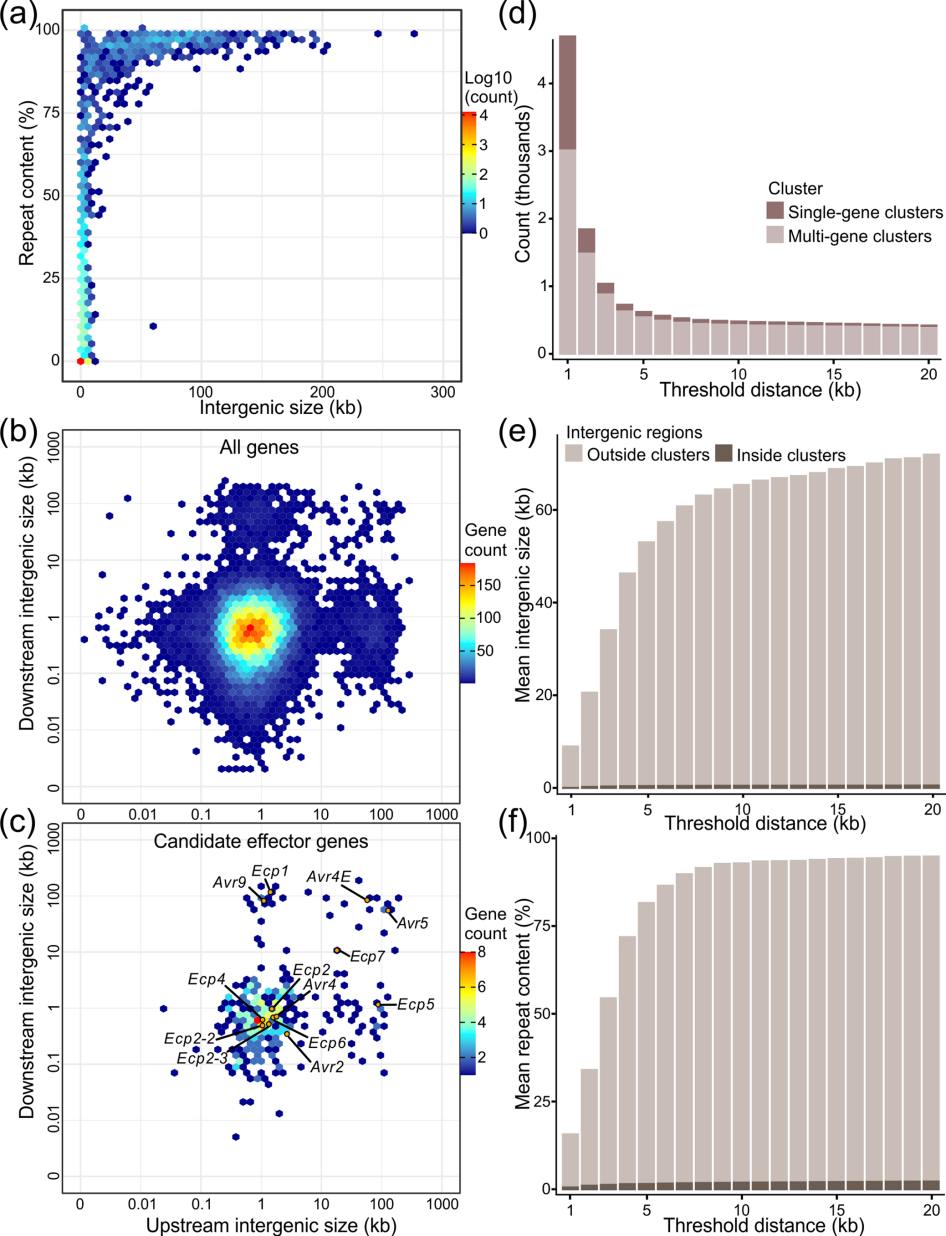
Compartmentalization of the genome of *Cladosporium fulvum* Race 5. (**a**) Heat map showing the number in log10 scale of intergenic sequences relative to their size and repeat content. This heat map shows that the genome of *C. fulvum* Race 5 is abundant in small, non-repetitive intergenic regions, whereas nearly all intergenic regions larger than 50 kb in size are almost entirely composed of repeats. This indicates that repeats in the genome of *C. fulvum* Race 5 are clustered and form long intergenic regions. (**b, c**) Heat maps showing the number of genes with the corresponding up- and downstream intergenic sizes on the x- and y-axis, respectively. The heat map in panel (**a**) includes all genes (*n*=14690), and the heat map in panel (**b**) includes only genes encoding candidate effectors (*n*=345). Previously described avirulence genes (*Avr*) and the extracellular protein encoding genes (*Ecp*) are indicated with points. The heat maps show that there are several candidate effector genes located in gene-sparse regions of the *C. fulvum* Race 5 genome and they typically have at least one neighbouring gene in close proximity, either up- or downstream of their coding sequence. However, the majority of candidate effector genes either follow the trend of all genes, i.e. located in gene-rich regions (e.g. *Avr2, Avr4*, *Ecp2, Ecp2-2, Ecp2-3, Ecp4,* and *Ecp6*), or are located next to some of the longest intergenic regions of the genome, i.e. in gene-sparse regions (e.g. *Avr4E, Avr5, Avr9, Ecp1, Ecp5*, and *Ecp7*). (**d, e, f**) Bar plots showing the statistics of gene clustering based on different threshold intergenic distance values (i.e. 1 to 20 kb) among genes within the same cluster. The bar plot in panel (**d**) shows the number of clusters identified, divided into single-gene clusters and clusters with more than one gene. The bar plots in panels (**e, f**) show the mean intergenic region size and percentage of repetitive DNA within intergenic regions outside clusters, i.e. flanking clusters, and inside clusters, respectively. The figures show that the genes of *C. fulvum* Race 5 can be organized into approximately 500 clusters separated by long intergenic regions that are rich in repeats, whereas intergenic regions inside clusters are poor in repeats.

Further examination of the organization of the chromosomes of *C. fulvum* Race 5 (Fig. 1) indicated that genes and repetitive regions are unevenly distributed on them, thus resulting in a parallel skewed distribution of the size of their intergenic regions. The median size of the intergenic regions is 646 bp, which is considerably small compared to the average size for the entire genome (3196 bp) and the size of the 5 % (*n*=735) longest intergenic regions, which ranges from 4127 bp to 278721 bp. Interestingly, in contrast to the large percentage of repetitive DNA in the genome of *C. fulvum* Race 5, the majority of the intergenic regions (*n*=12901; 87.8%) are free of repeats. However, from the 509 intergenic regions longer than 10 kb, 475 are composed of more than 80 % repeats, whereas all 243 intergenic regions longer than 60 kb are composed of at least 88.9 % repeats (Fig. 4a). These observations indicate that repeats are generally clustered instead of being dispersed throughout the genome of *C. fulvum* and that repeats are the major component of long intergenic regions.

An analysis of the sizes of the intergenic regions among all genes revealed a clear pattern of gene-rich and gene-sparse regions, in accordance with the two-speed genome model [24] (Fig. 4b). Several candidate effector genes were located in gene-sparse regions, including the previously described avirulence genes *Avr9*, *Avr4E,* and *Avr5,* and the extracellular protein encoding genes *Ecp1, Ecp5*, and *Ecp7,* all of which are flanked by some of the longest intergenic regions present in the genome (Fig. 4c). In contrast, *Avr2, Avr4*, *Ecp2, Ecp2-2, Ecp2-3, Ecp4,* and *Ecp6* are located in gene-rich regions. It should be noted that the coding sequence of *Avr5* is disrupted in *C. fulvum* Race 5 by a 2 bp deletion, which causes a frameshift that leads to a premature stop codon (Fig. S11). Genes present in gene-sparse regions typically have one long and one short intergenic region (Fig. 4b). This observation is consistent with a pattern of gene clustering in which, on the one hand, genes in gene-sparse regions are flanking clusters of genes and, on the other hand, the gene clusters are separated from each other by long intergenic regions. Based on this, the genes of *C. fulvum* Race 5 were organized into clusters, where a cluster is the largest set of consecutive genes in the genome such that the distance between any pair of adjacent genes is less than a defined threshold. Using different distance threshold values (i.e. between 1-to-20 kb), then the number of gene clusters identified in *C. fulvum* somewhat stabilizes to approximately 500 at threshold distances of over 5 kb (Fig. 4d, Table S12). For example, at the distance thresholds of 8 kb and 20 kb, there are 531 and 449 gene clusters, containing on average 27.7 and 32.7 genes, respectively. Notably, intergenic regions within gene clusters are typically short and have a low repetitive DNA content of less than 3 % on average. In contrast, intergenic regions between gene clusters are long and highly enriched with repetitive DNA of more than 90 % on average (Fig. 4e and f). This indicates that gene clusters are almost free of repetitive DNA, whereas genes that are flanking gene clusters are next to highly repetitive regions, and therefore likely more prone to mutations induced by transposon activity.

Although the majority (93.3%) of the genes have both intergenic regions shorter than 8 kb, there are 990 genes (6.7%) with intergenic regions longer than 8 kb up- or downstream of their coding sequence. Of these, 61 genes have intergenic regions longer than 8 kb at both sides, and thus correspond to single-gene clusters (i.e. isolated genes), whereas 929 genes are flanked by an intergenic region longer than 8 kb at only one side, and thus correspond to cluster-flanking genes. Among the 990 genes with long intergenic regions, only 391 (39.5 %) encode proteins with a Pfam domain in their primary structure, whereas the rest 599 (60.5 %) genes encode hypothetical proteins. An enrichment analysis based on a hypergeometric test showed that at an adjusted *p*-value<0.05, five Pfam domains are overrepresented in the subset of 391 genes. The three most significant of these Pfam domains (adjusted *p*-value<4E-4) are commonly associated with transposons and represent an endonuclease-reverse transcriptase domain (PF14529), a CHRromatin Organisation MOdifier domain (PF00385), and a reverse transcriptase domain (PF00078). Indeed, these domains are commonly present in predicted gene models that overlap masked regions of the genome (Table S13). The other two significant Pfam domains are a velvet factor domain (PF11754; adjusted *p*-value<8E-4) and a mycotoxin biosynthesis protein UstYa domain (PF11807; adjusted *p*-value=0.01). Finally, of the 990 genes present in gene-sparse regions, 142 encode secreted proteins, 66 of which are candidate effectors. A hypergeometric test showed that candidate effectors are significantly enriched (*p*-value=6.6E-15) within the set of 990 genes. In contrast, genes encoding for secreted proteins that are not candidate effectors and other gene categories were not significantly enriched (Table S14). It should be noted that, although gene-sparse regions are enriched for genes encoding candidate effectors, the majority (*n*=279, 80.9%) of candidate effectors are still present in gene clusters rather than gene-sparse regions (*n*=66, 19.1%).

### Gene duplication analysis reveals two identical copies of an *Avr3Lm*-like candidate effector gene in *C. fulvum* Race 5

**Fig. 5.**
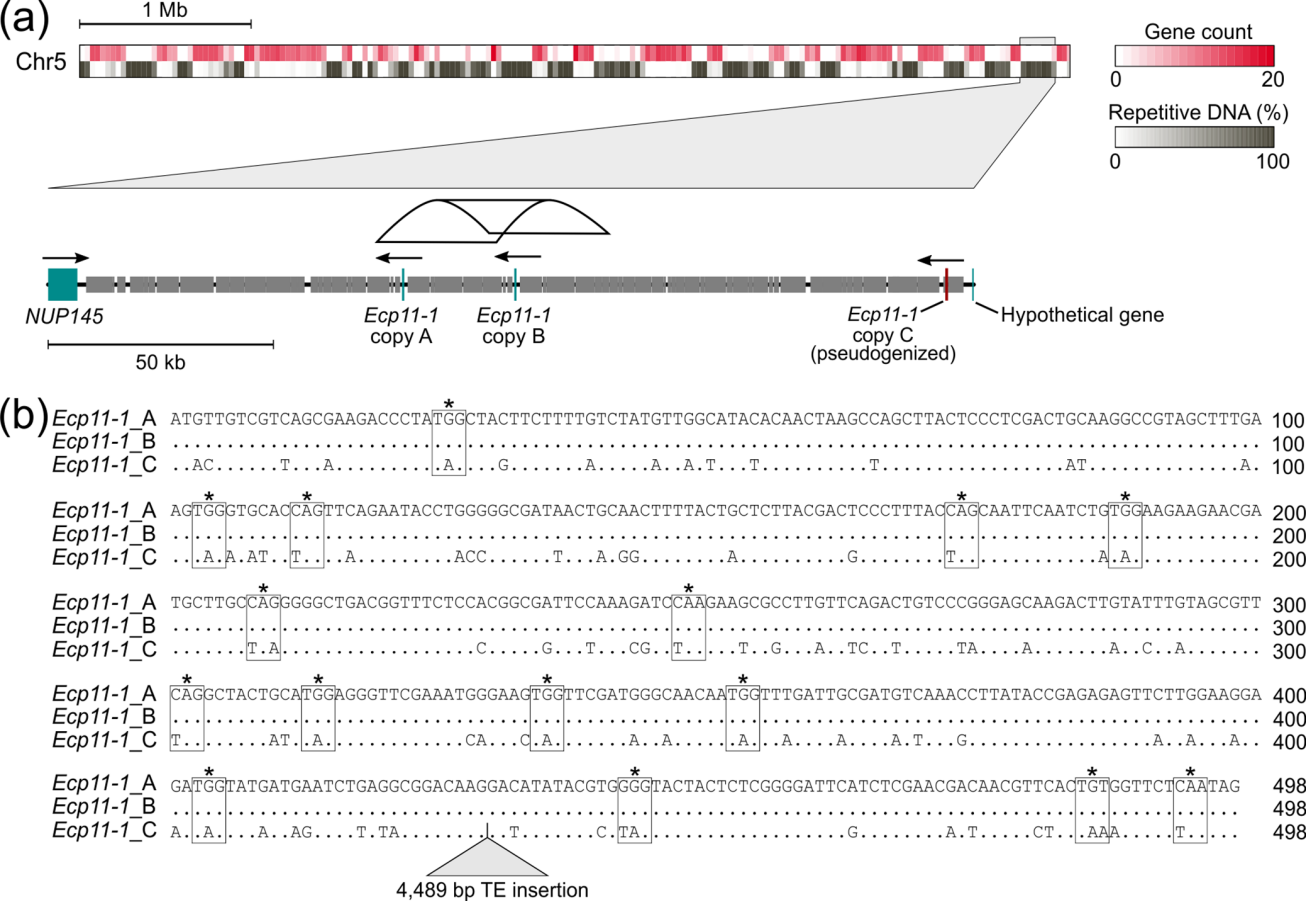
Segmental duplication of the gene encoding the candidate effector Ecp11-1 in *Cladosporium fulvum* Race 5. (**a**) A zoomed-in region near the end of chromosome 5 (Chr5). The zoomed-in region is 205.5 kb in size and shows the location of two identical copies of *Ecp11-1* (copy A and copy B) and an additional pseudogenized copy (copy C) of the gene, surrounded by repetitive DNA. Genes are represented as boxes with arrows indicating their transcriptional orientation. Repetitive regions are represented as smaller boxes. The duplicated segment that contains the two functional copies of *Ecp11-1* (copy A and copy B) is shown with a ribbon. The three copies of *Ecp11-1* are located between *NUP145*, encoding a component of the nuclear pore complex, and a hypothetical gene. (**b**) Global alignment of the coding sequences of the three *Ecp11-1* copies (copies A, B, and C). Codons mutated to a stop codon are indicated with boxes. The location of an insertion of a 4.5 kb transposable element in copy C is shown with a triangle.

One advantage long read-based assemblies have is that they allow identification of nearly identical copies of genes, likely caused by recent duplication events that would otherwise be collapsed in short read-based assemblies. To search for recently duplicated genes in the genome of *C. fulvum* Race 5, the coding sequences of the 14 690 genes predicted in it were clustered with *cd-hit-est,* using a minimum identity of 90 %. Twenty multi-gene clusters were identified, containing a total 59 genes (Table S15). Of the 20 multi-gene clusters, 12 clusters included genes similar to transposable elements, six clusters contained genes that encode hypothetical proteins, and one cluster included two genes similar to the proton-dependent oligopeptide transporter family, although one of the two copies is truncated and likely non-functional. The remaining cluster included two identical copies (copy A and copy B) of the candidate effector gene *Ecp11-1*, which is homologous (36.9 % identity at the amino acid level, e-value=4E-24) to the avirulence gene *AvrLm3* from the Dothideomycete *L. maculans* [105]. Notably, *Ecp11-1* is the only duplicated candidate effector gene retained in the genome of *C. fulvum* Race 5. Both copy A and copy B of *Ecp11-1* are intronless and are tandemly arranged in a repeat-rich region in Chr5 (Fig. 5a). To rule out the possibility that the two copies are the result of an assembly artefact, the PacBio reads were mapped to the genome and the region was confirmed to be free of misassemblies (Fig. S12). Based on a self-alignment performed with NUCmer, the identified duplication is 26.6 kb long, with 99.2 % alignment identity, and contains copy A and copy B of *Ecp11-1*, plus 5.4 kb and 20.6 kb down- and upstream of the two copies, respectively. A third copy (copy C) of *Ecp11-1* was identified 94.7 kb upstream of copy A (Fig. 5a). This third copy is predicted to be affected by RIP because its entire coding sequence resides within RIPped regions, and 70 (75 %) of the 93 point mutations compared to copy A and B are C:G to T:A transitions, which resulted in several premature stop codons. Therefore, *Ecp11-1* copy C is pseudogenized likely due to the accumulation of RIP-like mutations. In addition, a LINE-like transposable element of 4489 bp is inserted toward the 3′-end of the pseudogenized *Ecp11-1* (Fig. 5b). Overall, the small number of duplicated genes identified in the genome of *C. fulvum* Race 5 and the pseudogenization of one of the three copies of *Ecp11-1*, suggest that duplicated genes in *C. fulvum* are often lost, possibly due to the accumulation of mutations.

### Genome comparison between *C. fulvum* isolates Race 5 and 0WU exposes hundreds of genes missing in the previous reference genome assembly of *C. fulvum* 0WU

The first and so far only genome assembly of *C. fulvum* (isolate 0WU) was published nearly a decade ago [49] and since then, it has been used as a reference for comparative genome analyses. However, the assembly of isolate 0WU is highly fragmented into 4865 scaffolds. In order to compare the genomes of *C. fulvum* isolates Race 5 and 0WU, the scaffolds of isolate 0WU were split at gapped regions and the resulting 5715 contigs were then mapped to the genome of isolate Race 5. Out of the 5715 contigs, 5713 mapped to *C. fulvum* Race 5 chromosomes, covering 57086585 bp, or 84.9 % of the assembly. Coverage of most chromosomes was between 80 and 90 %, with almost all genes present in both isolates (Table S16). However, Chr12 was an exception, as the contigs of isolate 0WU covered only 53 % of this chromosome (Fig. S13a). In addition, when the gene sequences of *C. fulvum* Race 5 were queried with BLASTn (e-value <1e-5) against the genome assembly of isolate 0WU, then 352 genes had no BLASTn hit, and thus were missing from the assembly of isolate 0WU (Table S17). Notably, of the 352 genes, 348 are located in Chr12, corresponding to half (49.8%) of all predicted genes in this chromosome (Fig. S13a). The missing genes are unlikely dispensable because 74 of them are universal single copy orthologs (i.e. BUSCOs) that are conserved among Capnodiales. Among the 352 missing genes, there were 51 putative transporters, 30 secreted proteins, nine candidate effectors, eight CAZymes, and eight proteases (Table S17). Whole-genome sequencing data of *C. fulvum* 0WU is currently not publicly available to confirm the absence of these genes from its genome. However, upon analysis of *in vitro* RNA-seq data of *C. fulvum* 0WU (SRR1171044, SRR1171045, and SRR1171046), we observed that from the 352 genes missing, 241 show clear evidence of expression (transcripts per million [TPM]>2) in all three different RNA-seq datasets (Fig. S13b, Table S17). This indicates that most of the 352 genes are present in *C. fulvum* 0WU but they are missing in its reference genome assembly.

### Comparison of the *C. fulvum* Race 5 genome assembly with other Dothideomycetes indicates a core set of 13 mesosyntenic chromosomes

**Fig. 6.**
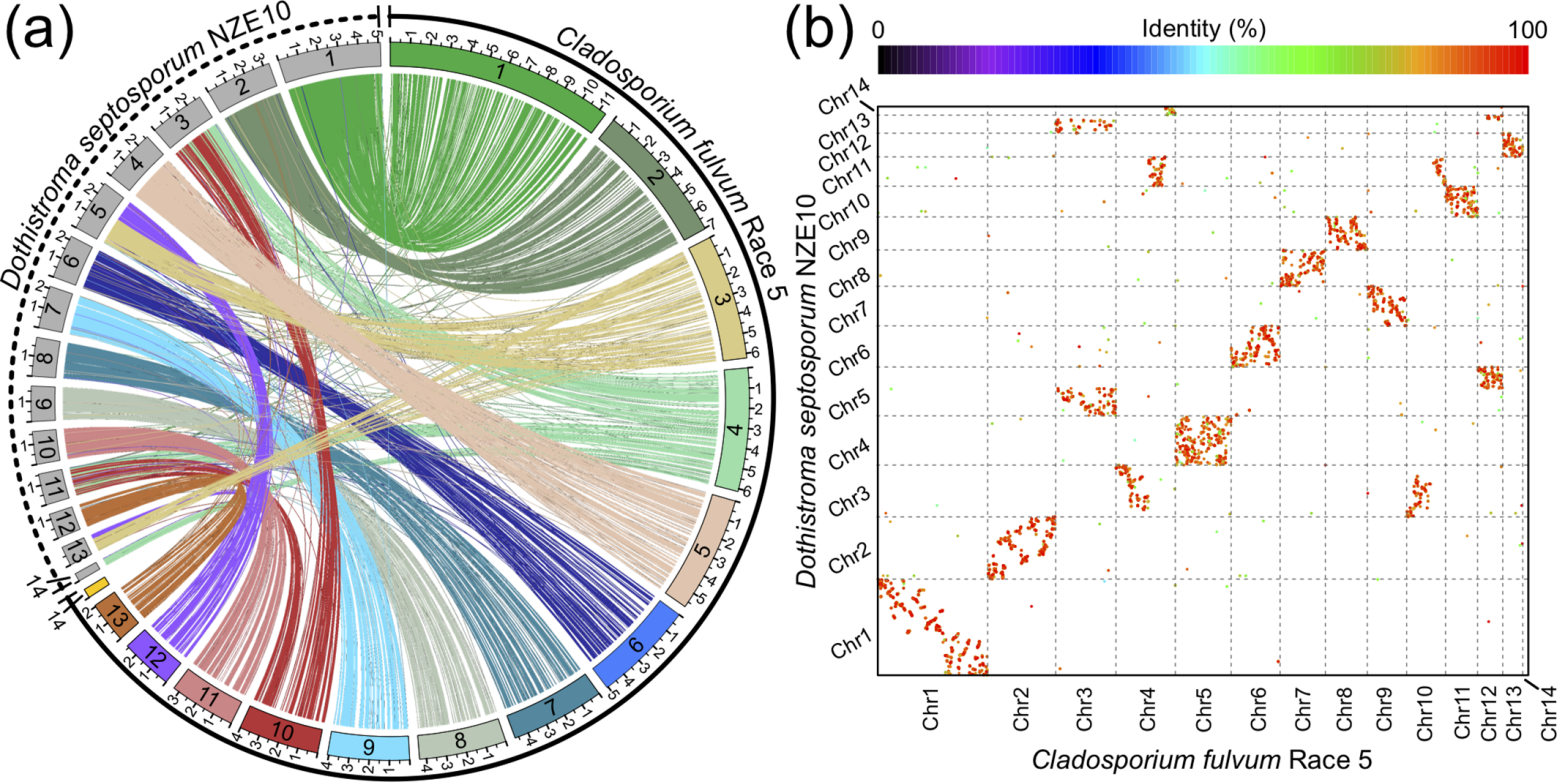
Mesosynteny between *Cladosporium fulvum* Race 5 and *Dothistroma septosporum* NZE10. The figure shows the whole-genome alignment produced with PROmer based on the six-frame translation of the genomes of *C. fulvum* Race 5 and of *D. septosporum* isolate NZE10. (**a**) Circos plot showing the collinearity between the chromosomes of the two species. Ribbons are based on nucleotide identity. (**b**) Dot-plot showing syntenic relations between the chromosomes of the two species. Chromosomes are numbered and dots are color-coded for percent nucleotide identity. The plots show a pattern of mesosynteny between *C. fulvum* Race 5 and *D. septosporum* NZE10, in which gene content is largely conserved within chromosomes, with few interchromosomal rearrangements, whereas gene order is not conserved. The alignment with the *D. septosporum* NZE10 genome produced high identity values, as shown in panel (**b**), which contrasts with the large difference in genome size compared to *C. fulvum* Race 5, as shown in the circos plot in panel (**a**). The mini-chromosome 14 (Chr14) of *C. fulvum* Race 5 had no matches with the genome of *D. septosporum* NZE10, supporting the hypothesis that this chromosome is dispensable. Chromosomes were named based on their size, from the longest to the smallest one, and are shown in scale in the circos plot, with tick labels indicating Mb. Whole-genome alignments of *C. fulvum* Race 5 with other Capnodiales are shown in Fig. S14.

The genome of the pine tree pathogen *D. septosporum*, a phylogenetically close relative of *C. fulvum*, has been previously assembled at chromosome-scale [49]. Both species have the same number of predicted chromosomes (*n*=14), but the size of their genomes varies markedly, ranging from 30.2 Mb for *D. septosporum* isolate NZE10 to 67.1 Mb for *C. fulvum* Race 5 (Fig. 6a). In order to compare the genome organization of the two species, their genomes were aligned with PROmer, which produced 17 337 aligned segments with an average length of 1000 bp and an average identity at the amino acid level of 73.8 % (Fig. 6a and b). The whole-genome alignment revealed a clear pattern of mesosynteny, consisting of a small number of inter-chromosomal translocations and a large number of intra-chromosomal rearrangements. This confirmed previous observations that suggested that the genomes of *C. fulvum* Race 5 and *D. septosporum* NZE10 are mesosyntenic [49]. This pattern is common within Dothideomycetes [22, 23] and indicates that gene content of both species is conserved within chromosomes, although the order of genes is not well conserved. Out of the 14 chromosomes present in each species, nine (i.e. Chr1, Chr2, Chr5, Chr6, Chr7, Chr8, Chr9, Chr11, and Chr13 with reference to the *C. fulvum* Race 5 chromosomes) have a one-to-one match, whereas of the five remaining chromosomes, four chromosomes of *C. fulvum* Race 5 (i.e. Chr3, Chr4, Chr10, and Chr12) match two or more chromosomes of *D. septosporum* NZE10. Finally, the mini-chromosome Chr14 of *C. fulvum* Race 5 has no matches to *D. septosporum* NZE10 chromosomes, whereas Chr14 of *D. septosporum* NZE10 matches Chr4 of *C. fulvum* Race 5 (Fig. 6a and b). A similar pattern of mesosynteny was also observed when the genome of *C. fulvum* was aligned with the genomes of the phylogenetically close relatives *Septoria musiva*, *Z. tritici*, *Pseudocercospora fijiensis,* and *Cercospora beticola* (Fig. S14). Notably, none of these has matches to Chr14 of *C. fulvum*, further supporting that this chromosome could be dispensable.

### The mini-chromosome Chr14 of *C. fulvum* is dispensable

The small size and low gene density of the mini-chromosome Chr14 led us to hypothesize that it might be a dispensable chromosome. To test this hypothesis, a collection of 24 isolates that were obtained from different locations around the world and in a span of over 40 years [52] was analysed for the presence or absence of Chr14. This was done using primers designed to capture eight genes located towards the 5′-end (one gene), middle (five genes), and 3′-end (two genes) of this mini-chromosome (Table S1 and Fig. S1a). PCR amplifications revealed that Chr14 was present in only five isolates, i.e. isolates Race 5, 0WU, 2, IMI Argent 358 077, and Turk 1a, out of the 24 isolates examined (Table 4, Fig. S1b). Interestingly, Chr14 was present in five out of the eight MAT1-2 isolates present in the collection, whereas none of the twelve MAT1-1 isolates had this chromosome. These results indicate that Chr14 is dispensable, and that it is more likely to be present in MAT1-2 than in MAT1-1 isolates. To further confirm dispensability of Chr14, DNA samples of isolates 2, IMI Argent 358 077, and Turk 1a, for which Chr14 was predicted to be present, were pooled (pool 1) and sequenced with Illumina technology. In addition, DNA samples for isolates IPO 2.4.8.9.11 Polen, IPO 249 France, and 2.5, for which Chr14 was predicted to be absent, were pooled (pool 2) and sequenced with Illumina technology as well. Sequenced reads were mapped to the genome of *C. fulvum* Race 5 at a mapping rate of 94%, and whole-genome coverage depths were estimated to be 59 × for pool 1 and 80 × for pool 2. Further coverage analysis revealed that 1.1 M reads from pool 1 and 0.26 M reads from pool 2 mapped to Chr14 (Fig. S15a). As expected, all genes predicted in Chr14 exhibited high levels of coverage of reads from pool 1 (Fig. S15b). In contrast, almost all predicted genes in Chr14 exhibited practically no coverage of reads from pool 2 (Fig. S15b). The only exception was the gene CLAFUR5_14645, which is duplicated in *C. fulvum* Race 5 with two identical copies, one in Chr14 and the other in Chr1. These results further support that Chr14 is indeed dispensable. The function of the genes in Chr14 remains elusive. Also, more isolates will have to be analysed for the presence/absence of Chr14 in their genome before a causal connection could be made between this mini-chromosome and one of the mating-type idiomorphs of *C. fulvum*.

**Table 4.** *Cladosporium fulvum* isolates [52] examined for the presence or absence of the mini-chromosome 14 (Chr14)

No.	Isolate	Race	Mating type	Origin	Year of collection	Presence of Chr14
1	2	2	MAT1-2	Netherlands	Unknown	Yes
2	2.4	2.4	MAT1-1	Netherlands	1971	No
3	2.4.5	2.4.5	MAT1-1	Netherlands	1977	No
4	2.4.5.9.11 IPO	2.4.5.9.11	MAT1-2	Netherlands	Unknown	No
5	2.4.5.9	2.4.5.9	MAT1-1	Netherlands	1980	No
6	2.4.8.11	2.4.8.11	MAT1-1	Netherlands	Unknown	No
7	2.4.9.11	2.4.9.11	MAT1-1	Poland	Unknown	No
8	IPO 2.4.5.9(60787)	2.4.5.9	MAT1-2	Netherlands	Unknown	No
9	IPO 2.4.8.9.11 Polen	2.4.8.9.11	MAT1-1	Poland	Unknown	No
10	2.5.9	2.5.9	MAT1-1	France	1987	No
11	4	4	MAT1-1	Netherlands	1971	No
12	IPO 249 France	2.4E	MAT1-1	France	Unknown	No
13	Can 38	4.4E	MAT1-1	USA	1962	No
14	IMI Argent 358 077	0	MAT1-2	Argentina	1991	Yes
15	Turk 1a	2	MAT1-2	Turkey	2005	Yes
16	L25	Unknown	Unknown	France	Unknown	No
17	2021–002	Unknown	Unknown	France	Unknown	No
18	18-A6	2.9	MAT1-2	Japan	Unknown	No
19	Can54b	2.3–1	Unknown	Unknown	Unknown	No
20	2.5	2.5	MAT1-1	Bulgaria	Unknown	No
21	Croatia 7	Unknown	Unknown	Croatia	2006	No
22	0WU	0	MAT1-2	Netherlands	Unknown	Yes
23	Race 4	4	MAT1-1	Netherlands	1971	No
24	Race 5	5	MAT1-2	France	1979	Yes

## Discussion

In this study, by combining long-read sequencing and Hi-C chromatin conformation capture data, we successfully produced a nearly complete genome assembly and a high-quality gene annotation for the TE-rich genome of *C. fulvum* Race 5. The assembly showed that the genome of this isolate consists of 13 core chromosomes and a dispensable mini-chromosome. The large percentage of Illumina reads that could be properly mapped to the final assembly and the high BUSCO completeness indicated that only a small portion of the genome, which may or may not contain genes, remains unassembled. The genome of *C. fulvum* Race 5 presented herein is a considerable improvement over the previous reference genome of *C. fulvum* 0WU [49]. Among the improvements worth noting, is the assembly of half of the genes in Chr12 that were somehow missed in the genome assembly of *C. fulvum* 0WU and of the long repetitive intergenic regions that previously could not be assembled. Moreover, the genome size of *C. fulvum* 0WU was underestimated during its sequencing, which translated into a reduced coverage of just 21-fold instead of the 32-fold coverage calculated previously, thus making it more likely that some genomic regions in isolate 0WU were not assembled due to low coverage. Such misassemblies caused by shallow sequencing coverage are expected to be randomly scattered throughout the genome and not to be concentrated in a particular chromosome, as was the case with Chr12. To this end, the reason why the gene space in Chr12 was unassembled in *C. fulvum* 0WU remains unknown.

The genomes of Dothideomycete fungi often display large and more than tenfold differences in size, with the increase in genome sizes typically instigated by their invasion by repetitive DNA and TEs [1]. High variability in repeat content is observed even among phylogenetically close-related species, as for example is the case in the *L. maculans-L. biglobosa* species complex, in which repeat content ranges from 3.9 % in the 30.2 Mb genome of *L. biglobosa* ‘canadensis’ strain J154, to 35.5 % in the 45.1 Mb genome of *L. maculans* ‘brassicae’ strain v23.1.3 [32]. In a similar way, species of *Pseudocercospora* spp. in the Sigatoka disease complex also exhibit large differences in genome size as a result of a high variation in their repetitive DNA content, which ranges from 35.7 % in the 53.79 Mb genome of *P. eumusae,* to 62.2 % in the 82.77 Mb genome of *P. musae* [106]. Contrasting genome sizes driven by differences in repeat and TE content were also observed between *C. fulvum* and *D. septosporum* [49]. Predicted TEs within the same family have an overall low divergence in *C. fulvum* Race 5, which is consistent with the hypothesis of a recent proliferation of TEs [107], possibly after divergence from *D. septosporum*. This could potentially explain why the genome of *C. fulvum* is markedly larger with expanded repeat content as compared to the genome of *D. septosporum* [49]. The overall low repeat divergence also supports recent proliferation of TEs. However, this observation contrasts with high levels of RIP mutations identified in the genome, which are expected to increase TE diversity. One explanation is that *C. fulvum* rarely undergoes sexual reproduction [49, 52], thus decreasing the speed at which RIP mutations accumulate over time. Further comparative genomic studies can provide insights into these contradictory observations.

Although TEs can potentially spread to any region of the genome, in fungal genomes they are typically unevenly distributed and tend to accumulate in gene-sparse regions where they often cluster with other TEs [7]. One explanation for this non-random distribution of TEs is that their disruptive effects upon insertion proximally to genes triggers purifying selection, which in turn will favour purging these elements from the population [108]. In plant pathogens, mutations or epigenetic modifications caused by the activity of TEs are major drivers of genetic variability associated with their evolution, genome plasticity, virulence, and host adaptation [7, 109, 110]. In this context, the uneven distribution of TEs across their genomes leads to their compartmentalization into TE-rich and TE-poor regions, in which TE-rich regions can accumulate mutations faster than TE-poor regions due to TE activity. This peculiar genome architecture is referred to as the ‘two-speed genome’ model [24, 25, 35] or the ‘dynamic compartmentalization’ [34]. In this model, TE-rich regions can provide a favourable environment to induce a fast evolutionary rate of virulence-related genes, which then can provide advantages for host adaptation. In agreement with the two-speed genome model, our analysis revealed that repetitive DNA sequences in the genome of *C. fulvum* Race 5 are clustered and form long, repeat-rich intergenic regions that intersperse gene-dense regions. Notably, gene-sparse regions were enriched with candidate effector genes, but not with other gene categories, suggesting that genome compartmentalization in *C. fulvum* could be a driver of virulence and adaptation to different host genotypes, although most of candidate effectors are not in gene-sparse, TE-rich regions. Indeed, the loss through complete gene deletion of certain effector genes from populations of *C. fulvum*, including for example of the *Avr4E*, *Avr5,* and *Avr9* genes, has been linked to overcoming their cognate resistance genes in tomato [111–113]. Previous genomic analysis indicated co-localization of effector genes (e.g., *Avr4E*, *Avr5*, and *Avr9*) and repetitive regions in *C. fulvum* 0WU [49]. This observation led to the hypothesis that the loss of these effectors is mediated by structural variations induced by neighbouring repeats [49, 102]. Our analysis confirmed that *Avr4E*, *Avr5*, and *Avr9* are located in repeat-rich regions, and further showed that they are flanked by some of the longest intergenic regions present in the genome of *C. fulvum* Race 5. In contrast, the *Avr2*, *Avr4*, *Ecp2*, and *Ecp4* effector genes for which selection pressure from cognate resistance genes in tomato led to the emergence of mutated alleles of these effectors with nucleotide substitutions or short INDELs [38], are located in repeat-poor, gene-rich regions. Collectively, these results support the hypothesis that complete gene deletions of effector genes in *C. fulvum* are induced by the presence of neighbouring repeats and that the location of candidate effectors in the genome of *C. fulvum* could potentially be used to foretell their population genetics and mode of evolution under selection pressure from cognate resistance genes in tomato.

Among the candidate effector genes flanked by repeat-rich regions is *Ecp11-1*, the only non-hypothetical gene in the genome of *C. fulvum* Race 5 with two identical copies. Previous studies have shown that Ecp11-1 triggers a hypersensitive response in various tomato accessions, indicating that it is likely an effector recognized by a cognate resistance protein in tomato [102]. Moreover, a recent study elucidated the crystal structure of Ecp11-1 and showed that it is also recognized by the oilseed rape resistance protein Rlm3 [114]. Ecp11-1 is predicted to belong to the so-called LARS (Leptosphaeria avirulence-suppressing) family of effectors, members of which have been detected in several Dothideomycetes [114]. *Leptosphaeria maculans* ‘brassicae’ has an expanded number of candidate LARS effectors (*n*=13), the majority of which are found grouped in three regions of the genome, most likely as a result of local duplication events [114]. In a similar way, the three *Ecp11-1* copies present in the genome of *C. fulvum* Race 5 are tandemly arranged on Chr5, possibly due to TE activity or genomic instability induced by the repeat-rich neighbouring regions. However, *Ecp11-1* copy C is pseudogenized by RIP-like mutations and a TE-like insertion in its coding sequence. This suggests that RIP mutations and TE activity can disrupt duplicated genes, thus preventing copy number variation in *C. fulvum*. Moreover, several polymorphisms were identified between the *Ecp11-1* copy C and copies A and B, which are intact and exhibit identical sequences. This suggests that the *Ecp11-1* copy C was likely derived from an older duplication event, whereas copies A and B were generated from a more recent duplication event.

Accessory (a.k.a. dispensable) chromosomes are present in widespread Eukaryotic taxa, including plants, animals, and fungi, and they typically provide no advantage to the host organism [12]. However, fungal accessory chromosomes receive particular attention because they may harbour genes associated with virulence or host adaptation [13, 19]. For example, the secreted in xylem (*SIX*) effector genes in *Fusarium oxysporum,* which are pathogenicity factors and drivers of host specificity among *formae speciales* of this species [16, 115–117], the *AvrLm11* effector gene of *L. maculans* that confers virulence on *Brassica napa* [118], and a pea pathogenicity cluster of *Fusarium solani* that detoxifies the phytoalexin pisatin and is required for virulence on pea [119], are just a few examples of virulence or pathogenicity factors encoded by genes present in accessory chromosomes. The Capnodiales *Z. tritici* has the largest number of accessory chromosomes (*n*=8) identified in fungal species so far [14] but their role still remains elusive and no clear association with pathogenicity has been established. However, a recent study indicated that their presence provides a small but significant increase in virulence [120]. To the best of our knowledge, the presence of accessory chromosomes among the Capnodiales has thus far been demonstrated only for *Zymoseptoria* spp. Here we show that the mini-chromosome Chr14 of *C. fulvum* Race 5 shows presence/absence variation among different isolates of the fungus and is therefore dispensable. Thus, at present, *C. fulvum* is just the second species of Capnodiales in which dispensable chromosomes are detected. It is currently unknown whether the presence of Chr14 in the genome of *C. fulvum* presents with any selective advantage to the fungus, as all the 25 genes present on it encode hypothetical proteins and no candidate effector genes were predicted to reside on this chromosome. However, evidence of gene flow between Chr14 and the core chromosomes were observed, with a duplicated hypothetical gene having one copy in Chr1 and another copy in Chr14 (Table S15). Gene flow between core and accessory compartments of the genome has been reported for the rice pathogen *Magnaporthe oryzae*, in which the effector genes *PWL2* and *BAS1* can be located on the core chromosomes or side-by-side in a dispensable chromosome [121]. This raises the possibility that Chr14 of *C. fulvum* acts as a reservoir that accelerates the evolution of genes by rapidly accumulating mutations via RIP leakage or other types of mutations induced by TE activity. This is supported by the fact that Chr14 is rich in repetitive DNA and heavily affected by RIP mutations with signs of abundant RIP leakage toward single-copy regions. However, more studies are required to provide insights about the function and importance of Chr14 in *C. fulvum*.

In summary, our work offers important and novel insights into the architecture and organization of the *C. fulvum* genome. Novel findings in this study include the presence of a dispensable mini-chromosome, the organization of genes into gene clusters that are flanked by repeat-rich regions in agreement with the two-speed model of evolution, and the separation between small and large chromosomes based on gene content. The genome of *C. fulvum* Race 5 provides a valuable resource for functional genomic and population genetic studies of this organism. It further highlights potential mechanisms underlying its adaptation to its tomato host by showing, among others, that the repeat-rich regions can serve as a cradle for genomic variability mediated by mutations and duplications induced via TE activity or genome instability. Future studies will focus on deciphering the importance for infections of the dispensable Chr14 and of genes with copy number variation in the genome of *C. fulvum*.

## Supplementary Data

Supplementary material 1

Supplementary material 2
