# Supplementary material for: A chromosome-scale genome assembly of the tomato pathogen Cladosporium fulvum reveals a compartmentalized genome architecture and the presence of a dispensable chromosome
Source: Microb Genom. 2022 Apr 26;8(4):000819. doi: 10.1099/mgen.0.000819 (PMC9453070; doi:10.1099/mgen.0.000819)
Supplement: Supplementary material 1 [file mgen-8-0819-s001.pdf]

Supplementary Materials

Title: **A chromosome-scale genome assembly of the tomato pathogen *Cladosporium fulvum* reveals a compartmentalized genome architecture and the presence of a dispensable chromosome.**

Authors/Affiliations: Alex Z. Zaccaron[1], Li-Hung Chen[1,#a], Anastasios Samaras[1], and Ioannis Stergiopoulos[1*]

[1] Department of Plant Pathology, University of California Davis, Davis, CA, USA.

[#a] Current address: Department of Plant Pathology, National Chung Hsing University, Taichung, Taiwan

*Correspondence: Ioannis Stergiopoulos: University of California Davis, Department of Plant Pathology, One Shield Avenue, Davis, CA 95616-8751, USA, Tel: +1-530-400-9802, email: istergiopoulos@ucdavis.edu.

Keywords: Accessory chromosome; Ecp11-1; effectors; gene duplication; repeat-induced point mutation; fungal pathogen evolution; two-speed genome;

Supplementary Tables

(provided in a separate Excel file)

Table S1. Primers designed to capture genes located in the dispensable chromosome 14 (Chr14) of *Cladosporium fulvum* Race 5. A map with the location of these primers is shown in Fig S1a.

Table S2. Contigs assembled with Canu using sequenced PacBio reads of *Cladosporium fulvum* Race 5. Assembled bacterial contigs contained high GC content, and were discarded from the final assembly. Other smaller contigs were also discarded, as they matched the mitochondrial genome of *C. fulvum*, were contained within other contigs, or were assembled from a single PacBio read. Discarded contigs are highlighted.

Table S3. Summary of estimated abundance of transposable elements in the genome of *Cladosporium fulvum* Race 5. Custom repeat libraries were generated with RepeatModeler v1 and v2, and then used to mask the genome with RepeatMasker. The output of RepeatMasker was parsed with the script parseRM.pl (https://github.com/4ureliek/Parsing-RepeatMasker-Outputs), which counted the number of masked bases as reported in this table. In order to more accurately estimate the percentage of repeats of each class or family of transposable elements, bases masked twice (i.e. overlapping repeats) were not considered.

Table S4. Statistics of Repeat-Induced Point (RIP) mutations in the chromosomes of *Cladosporium fulvum* Race 5. The chromosomes were analyzed using a 1 kb sliding window and a step size of 500 bp. Windows were considered RIPped if the substrate index value (CpA + TpG)/(ApC + GpT) was ≤ 0.75, product index value (TpA/ApT) was ≥ 1.1, and composite index value (TpA/ApT) − ([CpA + TpG]/[ApC + GpT]) was ≥ 0.01. Single-copy windows were considered as RIPed windows when they had no secondary BLASTn hit against the *C. fulvum* Race 5 genome (e-value < 1E-20, identity > 50%, and query coverage > 20%). The last three columns show (i) the estimated percentage of RIPped regions determined as the total percentage to RIPped windows, (ii) the estimated percentage of repetitive regions RIPped determined as the percentage of repeat-masked bases that overlap RIPped windows, and (iii) the estimated percentage of single-copy regions RIPped determined as the percentage of unmasked bases within single-copy RIPped windows.

Table S5. Genes in the genome of *Cladosporium fulvum* Race 5 encoding key enzymes for secondary metabolism. These key enzymes are classified into non-ribosomal peptide synthetases (NRPS), type 1 polyketide synthases (T1PKS), and terpene synthases (Terpene). NRPS-like represent fragments of NRPS genes.

Table S6. Genes in the genome of *Cladosporium fulvum* Race 5 encoding predicted carbohydrate-active enzymes (CAZymes). CAZymes are classified into six major classes, i.e., auxiliary activity (AA), carbohydrate-binding module (CBM), carbohydrate esterase (CE), glycoside hydrolase (GH), glycosyltransferase (GT), and polysaccharide lyase (PL). Predicted secreted proteins are indicated in the third column.

Table S7. Genes in the genome of *Cladosporium fulvum* Race 5 encoding predicted proteases. Proteases are classified into aspartic (A), cysteine (C), metallo (M), asparagine (N), serine (S), threonine (T), and inhibitory (I) proteases. Proteases were identified based on BLASTp searches (e-

value < 1E-10) against the MEROPS database. Predicted secreted proteins are indicated in the third column.

Table S8. Genes in the genome of *Cladosporium fulvum* Race 5 encoding putative transporters. Transporters were identified based on BLASTp searches (e-value < 1E-10) against the Transporter Classification Database (TCDB).

Table S9. Secreted proteins and candidate effectors in the genome of *Cladosporium fulvum* Race 5. The table shows all proteins containing a predicted signal peptide (SP), their size (amino acids), prediction of EffectorP, number of transmembrane (TM) domains in the mature protein, predicted GPI-anchor (PFrate ≤ 0.005 means probable GPI-anchor), number and percentage of cysteines residues, name of homologous candidate effector previously described, and classification of the proteins into predicted effector (SSP) or non-effector (noSSP).

Table S10. Genes located in subtelomeric regions in the genome of *Cladosporium fulvum* Race 5.

Table S11. Over- and under-representation of different gene categories in subtelomeric regions in the genome of *Cladosporium fulvum* Race 5. Gene densities (count per Mb) for the whole genome and within subtelomeric regions (i.e. within 25 kb of telomeric repeats) are shown. There was a total of 143 genes presented within subtelomeric regions. The columns 4 to 7 show the number of genes from the specific category located in subtelomeric regions, number of genes in subtelomeric regions that do not belong to the specific gene category, total number of genes from the specific category in the genome, and total number of genes in the genome that do not belong to the specific category. These numbers were used in the phyper function within R to perform hypergeometric tests for over- and under-representation. Resulting p-values are shown in the last columns.

Table S12. Summary of gene clustering in the genome of *Cladosporium fulvum* Race 5. Genes were clustered based on different maximum threshold distances of 1 to 20 kb. For each threshold distance, the table shows the total number of gene clusters and the number of clusters with only one gene or more than one gene. Other fields present average numbers for the clusters identified, including average cluster size, average number of genes, and average size and repeat content of intergenic regions inside out outside clusters.

Table S13. Predicted genes in the genome of *Cladosporium fulvum* Race 5 that overlap with masked repetitive regions. The table shows genes that overlap more than 25% of their sequences with regions of the genome masked with RepeatMasker based on repeat libraries produced with RepeatModeler v1.0.11 (RM1) and RepeatModeler v2.0.2 (RM2). The percentages of the gene regions that overlap with masked regions are shown, as well as conserved PFAM domains found in the genes. The table shows that the genes that overlap with masked regions typically contain conserved domains commonly found in transposable elements.

Table S14. Statistical analysis to test enrichment of specific gene categories within gene-sparse regions of the genome of *Cladosporium fulvum* Race 5. A total of 990 genes with up- or downstream intergenic size of at least 8 kb were considered in genes-sparse regions. The columns 2 to 5 show the number of genes from the specific category located in gene-sparse regions, number of genes in gene-sparse regions that do not belong to the specific gene category, total number of genes from the specific category in the genome, and total number of genes in the genome that do not belong to

the specific category. These numbers were used in the *phyper* function within R to perform a hypergeometric test. Resulting p-values are shown in the last column.

Table S15. Putative recently duplicated genes in *Cladosporium fulvum* Race 5. Gene clusters identified with cd-hit-test by grouping coding sequences with at least 90% nucleotide identity. Representative sequences of the clusters are indicated with an asterisk.

Table S16. Genes from the genome of *Cladosporium fulvum* Race 5 and their corresponding ortholog in the genome of *C. fulvum* isolate 0WU (JGI).

Table S17. Genes present in the genome of *Cladosporium fulvum* Race 5 but missing the assembly of *C. fulvum* isolate 0WU. Location of the genes in the genome and functional descriptions are shown. Genes encoding universal single-copy orthologs conserved in species of Capnodiales are indicated with the respective BUSCO ID. Genes encoding putative transporters are indicated with the respective transporter family. Genes encoding Carbohydrate-active enzymes (CAZymes) and proteases are indicated with the respective CAZyme and protease family. Genes encoding secreted proteins or candidate effectors are also shown. Expression values in transcripts per million are shown in the last three columns for three different data sets of *C. fulvum* 0WU grown *in vitro*. Most missing genes have evidence of expression, indicating that they were misassembled in the genome of isolate 0WU.

Supplementary Figures

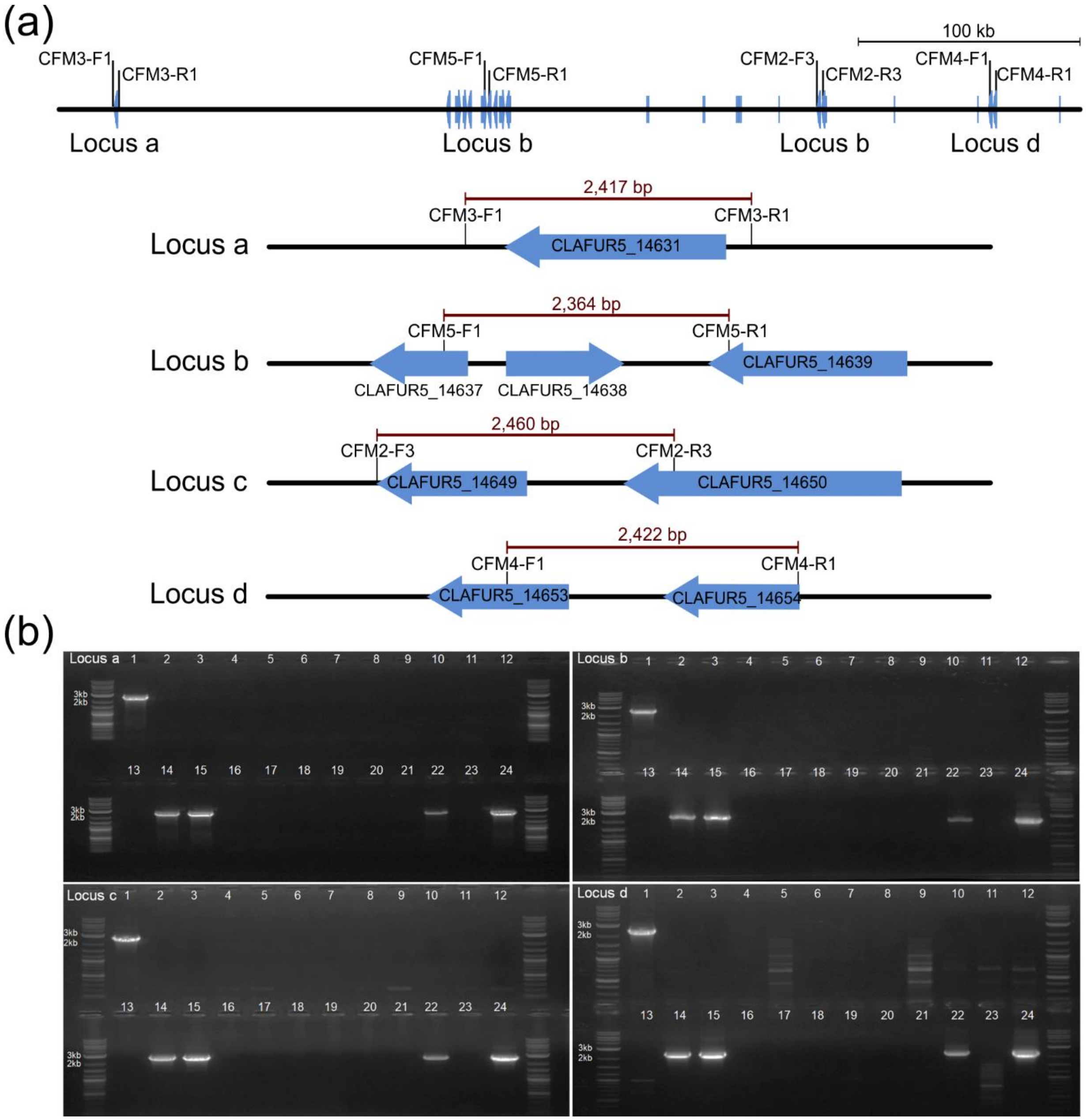

Fig. S1. Presence/absence variation of the mini-chromosome 14 (Chr14) in 24 isolates of *Cladosporium fulvum*. (a) Location of the primers used to determine the presence or absence of Chr14 among the 24 *C. fulvum* isolates examined in this study (Table 4). The figure shows an overview of the entire Chr14 as well as zoomed-in regions where primers are located. The regions (i.e. loci) were named a, b, c, and d. Predicted genes are represented as arrows. Primer sequences are shown in Table S1. (b) Gel electrophoresis analysis of polymerase chain reaction (PCR) amplicons obtained using the primer combinations in regions a, b, c, and d. The figure shows that only five isolates, i.e. isolates 1, 14, 15, 22, and 14, had the chromosome Chr14. Ladder is a 1kb Plus (New England Biolabs).

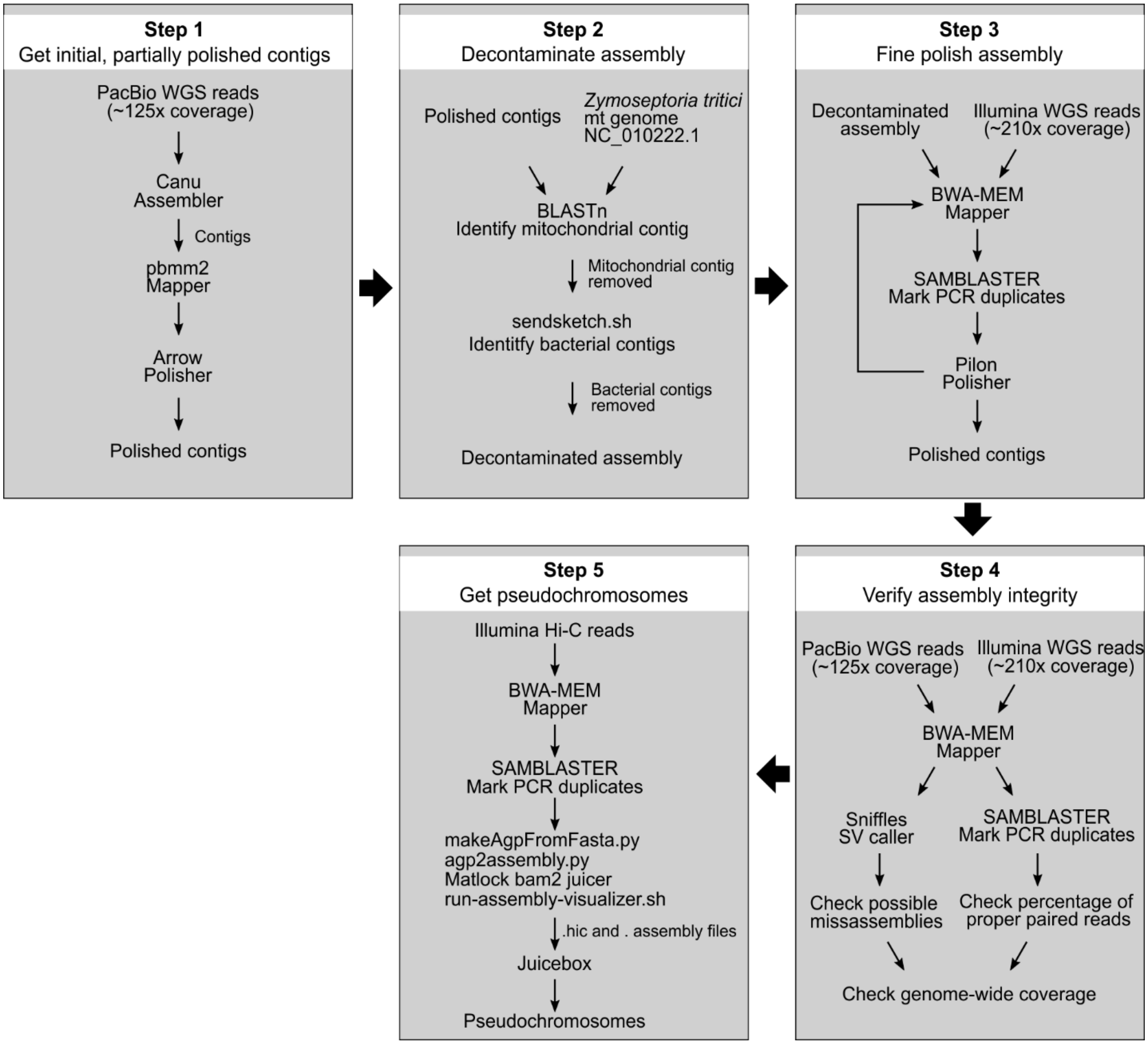

Fig. S2. Workflow used to assemble the genome of *Cladosporium fulvum* Race 5. The workflow has five major steps. First, contigs are assembled with Canu based on PacBio reads, followed by an initial round of polishing performed with Arrow. This round of polishing performed 7,347 changes, most of which (n = 7,020) were short INDELs of at most 13 bp. Assembly decontamination is performed in step 2. Specifically, from the 43 assembled contigs, 24 of them with a total size of 12.9 Mb matched bacterial genomes. These 24 contigs were then removed along with another four contigs of a total size of 186.4 kb as they were either contained within other contigs or they were formed from a single PacBio read. Another contig of 179.1 kb in size containing the mitochondrial genome of *C. fulvum* was also removed in this step. In step 3, the decontaminated assembly is fine polished with Illumina reads. This step was performed twice. In the first round, 692 changes were performed, whereas only four changes were performed in the second round. In step 4, potential misassemblies are identified by mapping the PacBio reads to the contigs and calling structural variants with Sniffles. Collapsed regions in the assembly are identified by mapping the PacBio and Illumina reads to the contigs, and verifying regions with abnormally high coverage. Finally, in step 5 Hi-C reads are mapped to the contigs and pseudochromosomes are identified.

Chr2:6,902,000-6,909,001

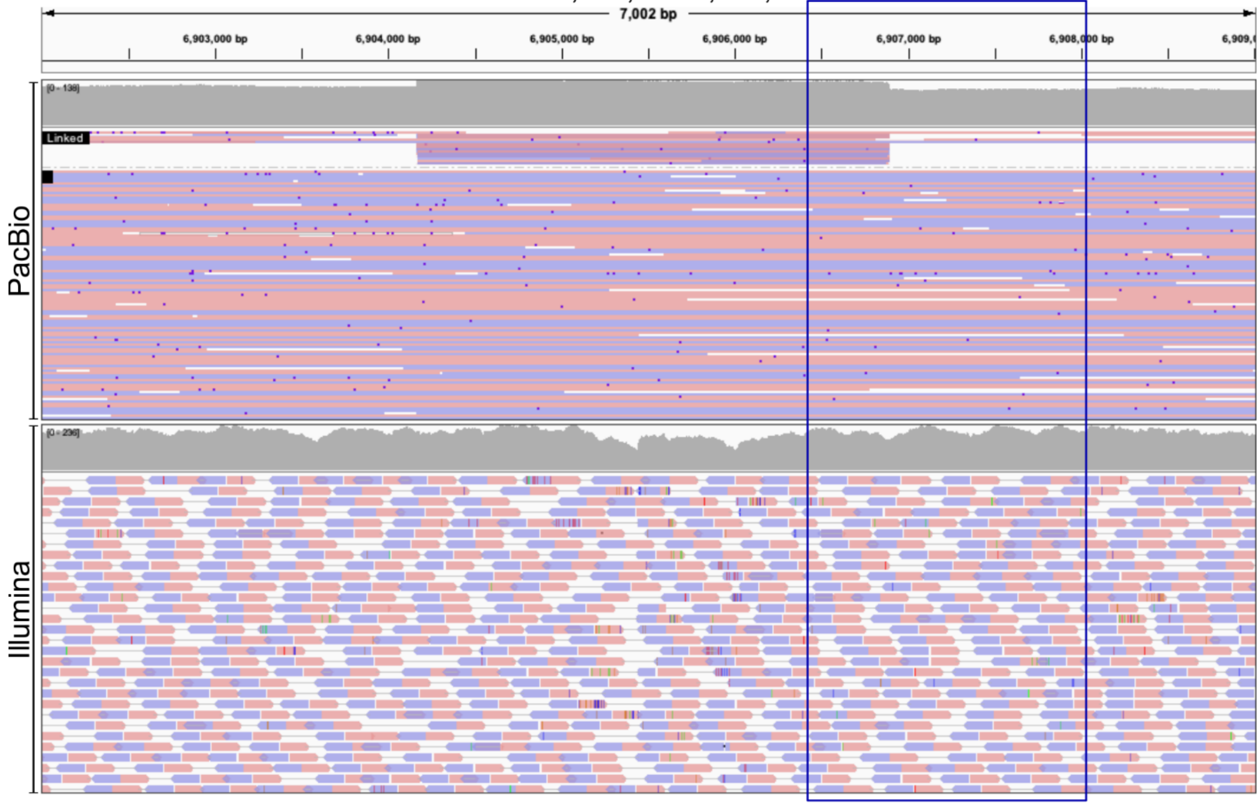

Chr8:1,993,000-1,999,001

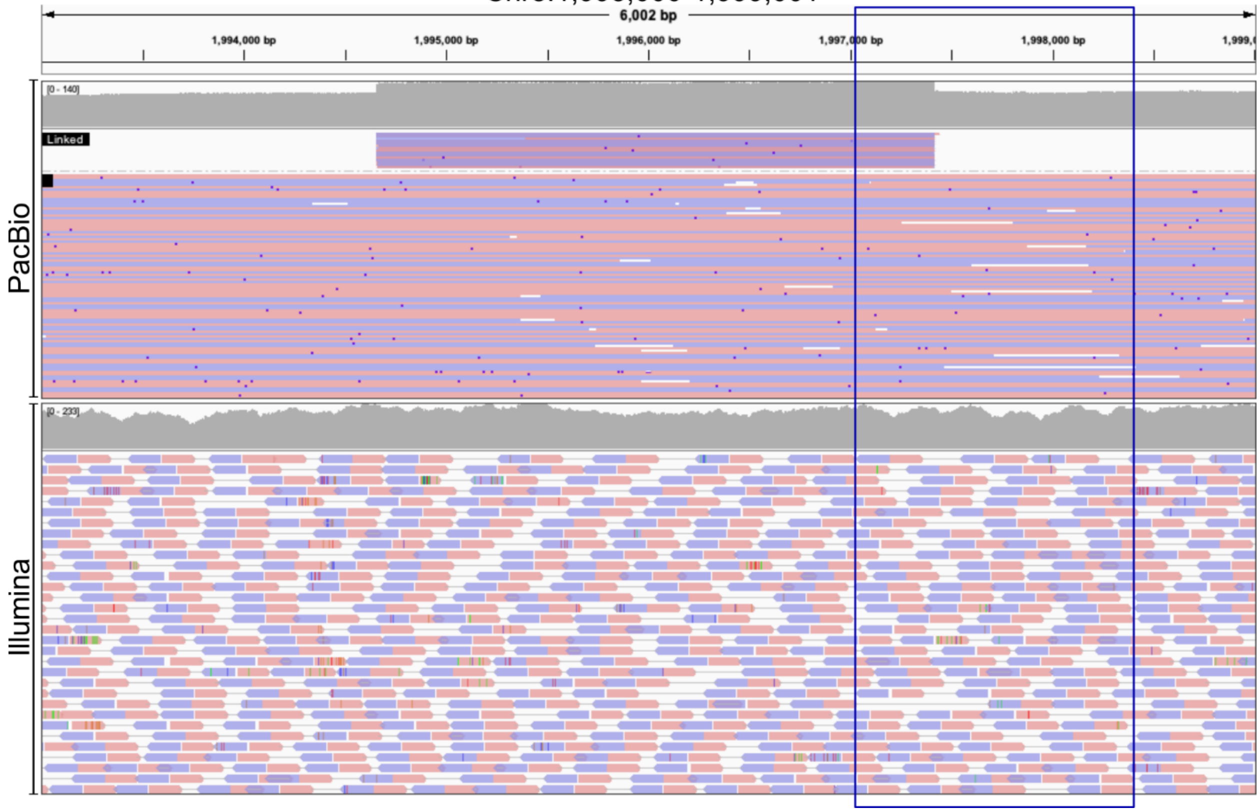

Fig. S3: Two putative misassembled regions weakly supported by raw sequencing data in the genome assembly of *Cladosporium fulvum* Race 5. Coverage of PacBio and Illumina reads are show for both regions. For PacBio, reads with supplementary alignments are shown at the top and supplementary alignments of the same read are linked (links are not visible because supplementary alignments of the same read have overlapping mapping coordinates). Each of these PacBio reads shown at the top in both regions have exactly one supplementary alignment mapped to the same location with opposite orientation as the primary alignment. These PacBio reads are likely the reason that Sniffles predicted an inverted tandem duplication in both regions (approximate location indicated with square boxes). However, most of the PacBio reads as well as the Illumina reads do no support the presence of inverted tandem duplications or other type of misassembly, because most PacBio reads span the entire regions and almost all Illumina reads mapped to these two regions are properly paired and uniquely mapped (mapping quality = 60).

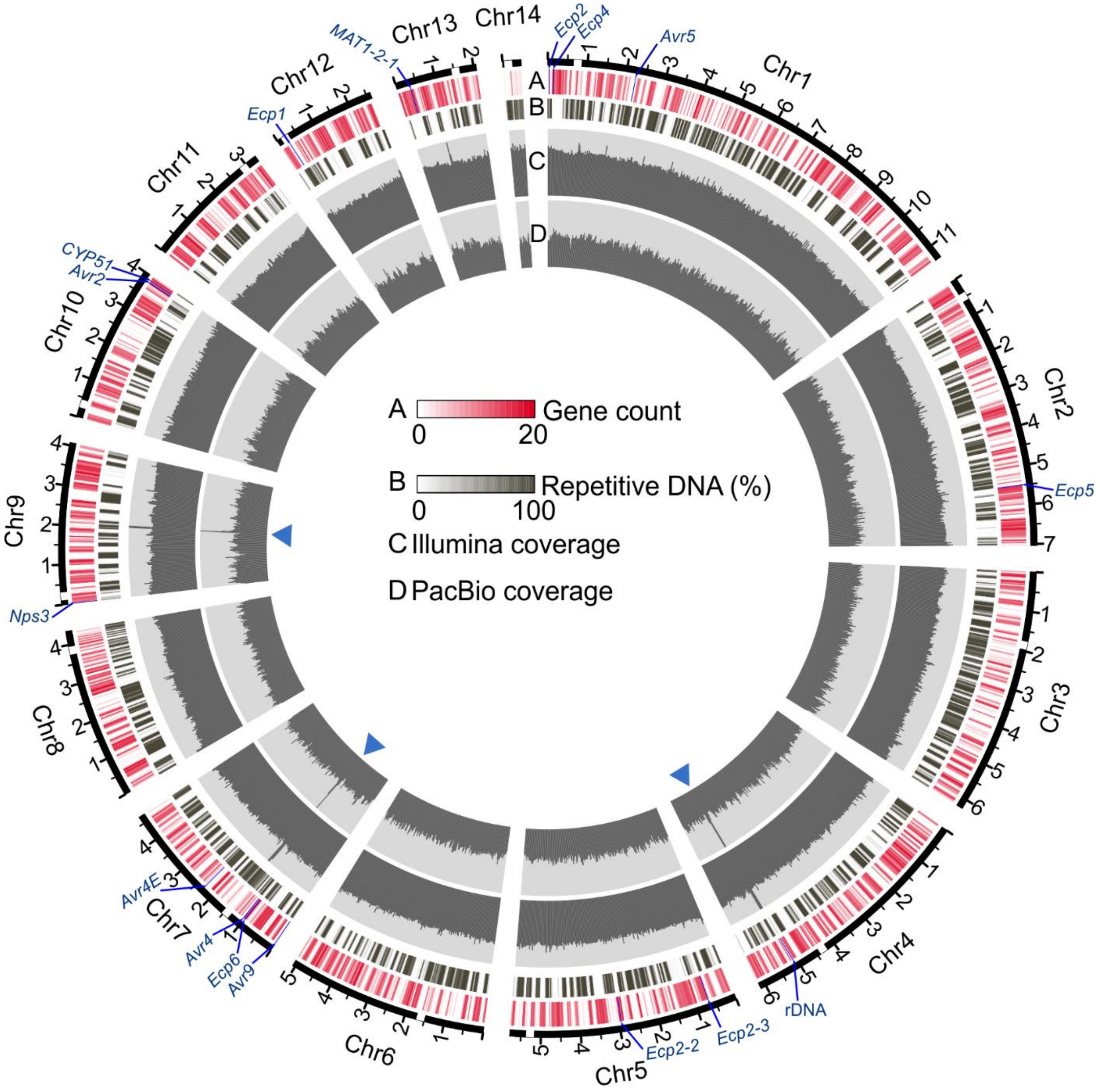

Fig. S4. Integrity of the assembly of *Cladosporium fulvum* Race 5. (a) Two genome locations where Sniffles predicted inverted tandem duplications (approximate location with square boxes) based on the PacBio reads, suggesting possible misassemblies. The PacBio and Illumina reads that mapped to these regions are shown in grey, with soft clipped regions indicated in color-coded base pairs (i.e., adenine in green, cytosine in blue, guanine in brown, and thymine in red). Some PacBio reads were only partially mapped to these regions but the mapped Illumina reads do not suggest problems with the assembly in these regions. (b) Circos plot showing the assembled chromosomes. The two outermost tracks show the gene and repetitive DNA content, respectively. The histograms in the two innermost tracks represent the coverage (0x to 200x) of the chromosomes by the generated Illumina and PacBio reads. The figure shows three possible collapsed regions indicated by triangles in the innermost track, where read coverage surpasses twice the median for the entire genome. Illumina reads marked as 'PCR duplicated' in the sequencing data were not considered to plotting the histogram. Illumina and PacBio coverage were calculated using a sliding window of 30 kb.

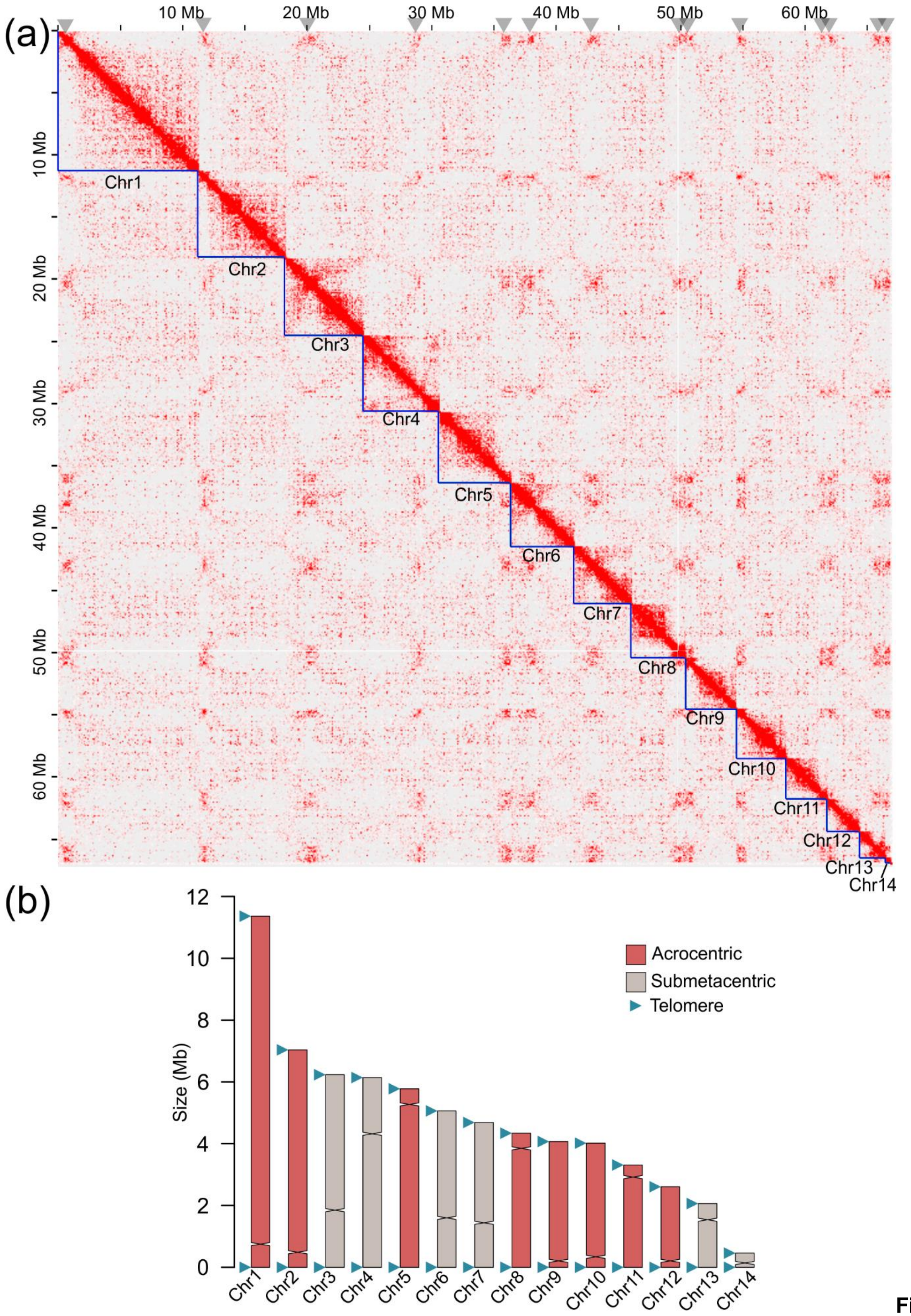
(a)
10 Mb
20 Mb
30 Mb
40 Mb
50 Mb
60 Mb
Chr1
Chr2
Chr3
Chr4
Chr5
Chr6
Chr7
Chr8
Chr9
Chr10
Chr11
Chr12
Chr13
Chr14
(b)
Size (Mb)
0
2
4
6
8
10
12
Acrocentric
Submetacentric
Telomere

Fig.

S5. Chromosomes of *Cladosporium fulvum* Race 5 identified based on an analysis of Hi-C data. (a) Heat map showing *all-versus-all* interaction frequency across the genome. The predicted 14 chromosomes are highlighted with blue lines. Putative centromeric regions are indicated with triangles at the top of the figure. In fungi, centromeric regions typically have high inter-chromosomal interaction frequency. The heat map was visualized and exported with Juicebox v1.11.08 at resolution (i.e. bin size) of 100 kb. (b) Classification of the 14 chromosomes of *C. fulvum* Race 5 into submetacentric or acrocentric, based on the putative location of the centromeres. Submetacentric chromosomes have a ratio of long arm and short arm sizes between 1 and 3, and acrocentric chromosomes have a ratio of long arm and short arm sizes greater than 7. Presence of telomeric repeats at the chromosomes' immediate ends is indicated by triangles.

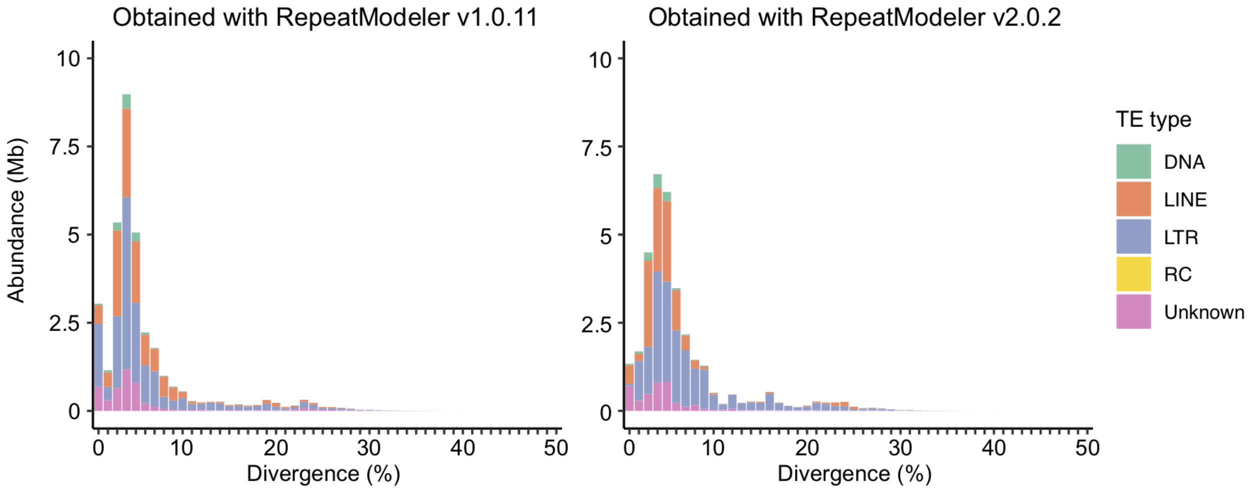

Fig S6. RepeatModeler v1 and RepeatModeler v2 produce similar results using the *Cladosporium fulvum* Race 5 genome data as input, and support an overall low repeat divergence in this pathogen. Bar plot showing the number of bases (y-axis) covered by predicted transposable elements (TEs) of different (sub)classes, i.e., DNA transposons (DNA), long interspersed nuclear elements (LINE), long terminal repeats (LTR), rolling-circles (RC), and unclassified TEs. The x-axis shows the divergence of repeats from the consensus sequences. The figure shows that the genome of *C. fulvum* Race 5 is abundant in repeats with an overall low divergence.

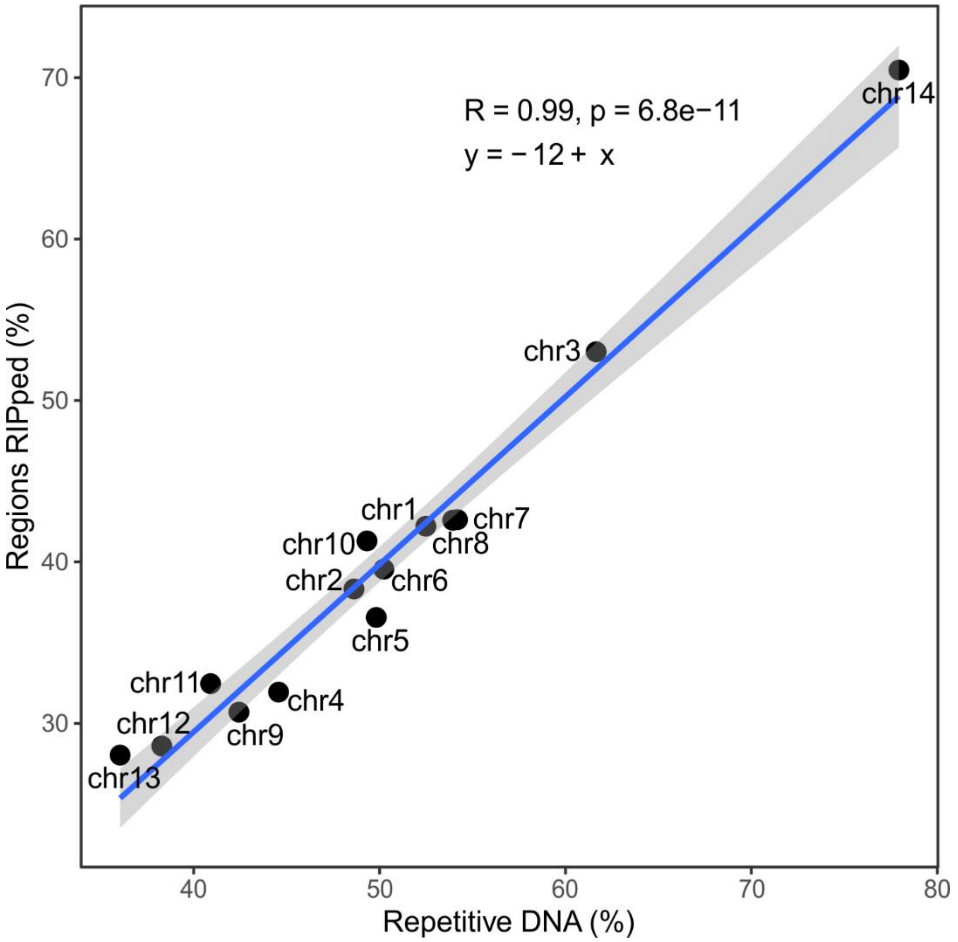

Fig. S7. Correlation between repetitive DNA content and percentage of regions affected by Repeat-Induced Point (RIP) mutations in the chromosomes of *Cladosporium fulvum* Race 5. The scatter plot shows a positive correlation between repetitive DNA content and the percentage of regions affected by RIP. A regression line is shown in blue and was determined with the *geom_smooth* function from the R package ggplot2, utilizing the lm method. Dark areas represent confidence intervals (95%). Correlation coefficient, p-value and the equation of the regression line are shown at the top of the plot.

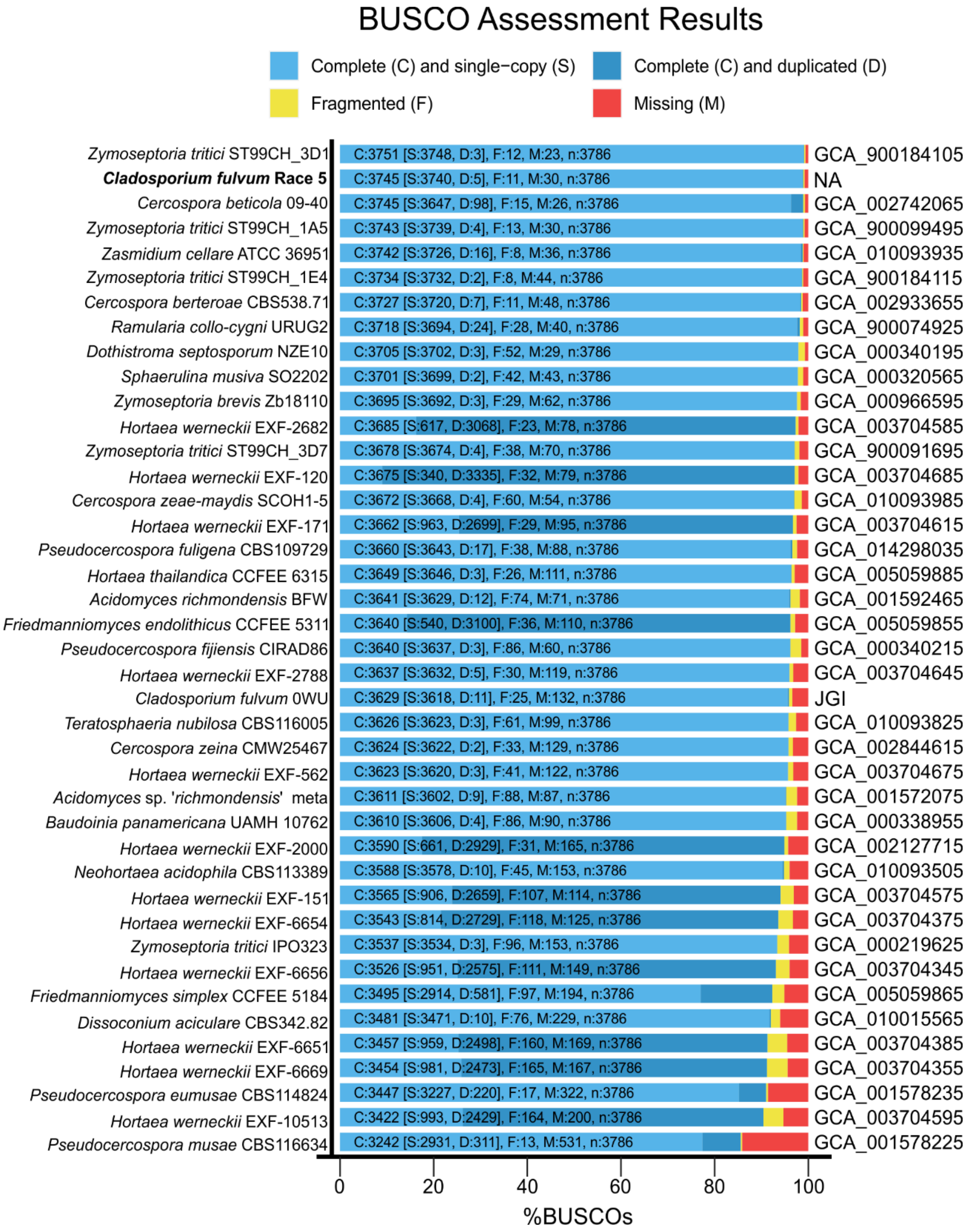

Fig. S8. Comparison of BUSCO completeness of *Cladosporium fulvum* Race 5 and other annotated genomes of Capnodiales. Genomes were ordered by estimated completeness (i.e., complete single copy plus complete duplicated BUSCOs). Genome accession numbers are shown on the right-hand side, except for *C. fulvum* Race 5 and *C. fulvum* 0WU (obtained from JGI MycoCosm). Values were obtained with BUSCO v5.2.1 in protein mode, using the Dothideomycetes_odb10 (2020-08-05) data base containing 3,786 BUSCOs as reference. Genomes were obtained from NCBI (2021-07-28).

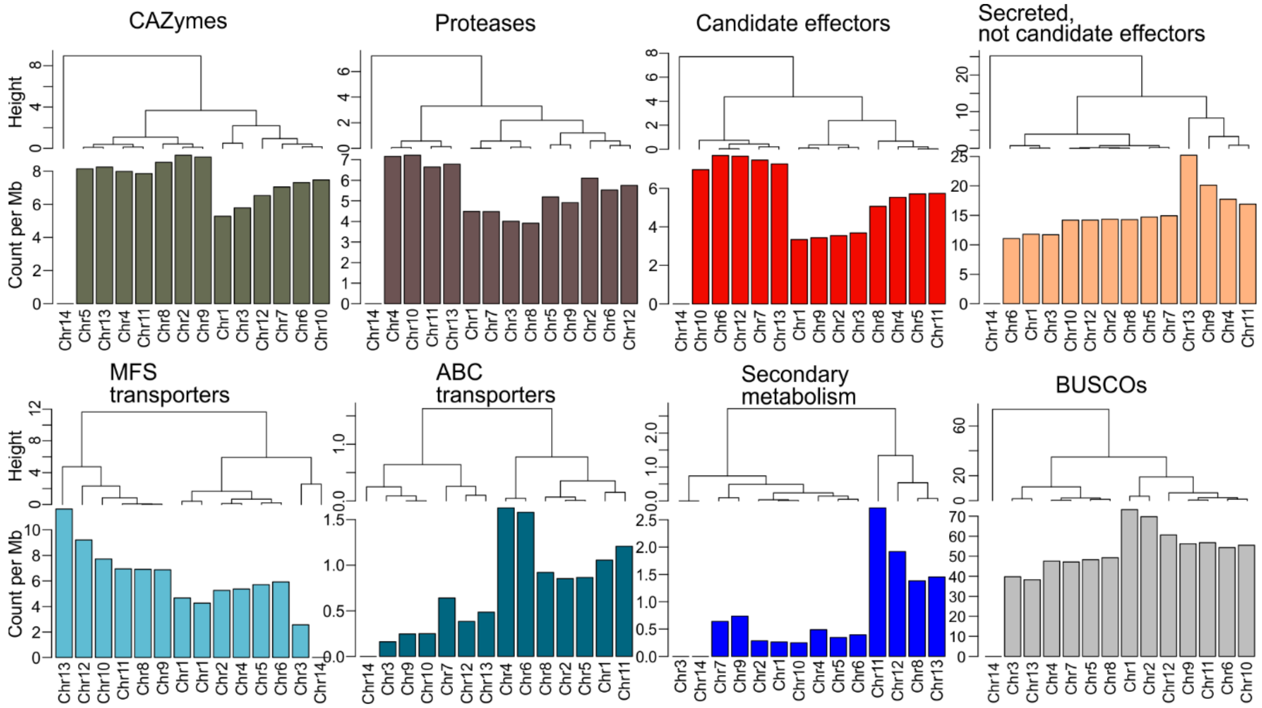

Fig. S9. Distribution of various gene categories within the chromosomes of *Cladosporium fulvum* Race 5. Bar plots showing hierarchical clustering of chromosomes based on gene densities of specific gene categories. Hierarchical clustering was performed using the *complete* method based on the Euclidean distances.

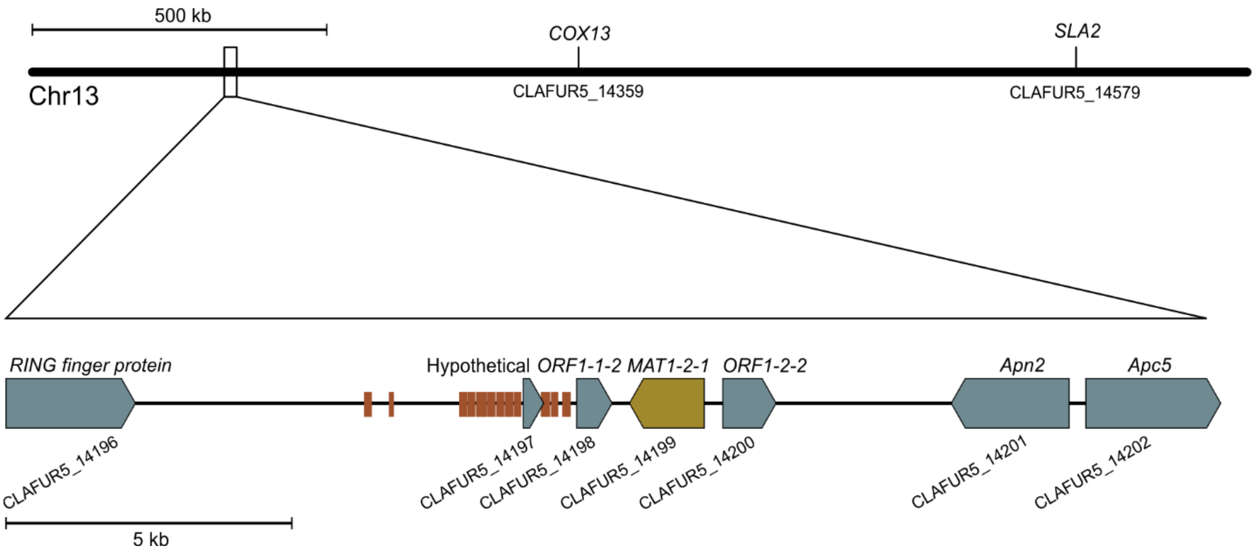

Fig. S10. Organization of the *MAT1-2* mating locus present in *Cladosporium fulvum* Race 5. The figure shows the location of the *MAT1-2-1* gene in Chr13. Two genes, named *ORF1-1-2* and *ORF1-2-2,* encoding hypothetical proteins flank *MAT1-2-1*. Three genes, i.e. *Apn2, SLA2,* and *COX13*, are typically located near *MAT* genes in Ascomycetes. However, only *Apn2* was near *MAT1-2-1* in *C. fulvum*. The location of *SLA2* and *COX13* in Chr13 are indicated with vertical lines. Other genes surrounding the MAT locus in *C. fulvum* encode a putative RING finger protein, a hypothetical protein, and the putative subunit 5 of the anaphase-promoting complex (Apc5). Repetitive regions are indicated with rectangles.

(a)

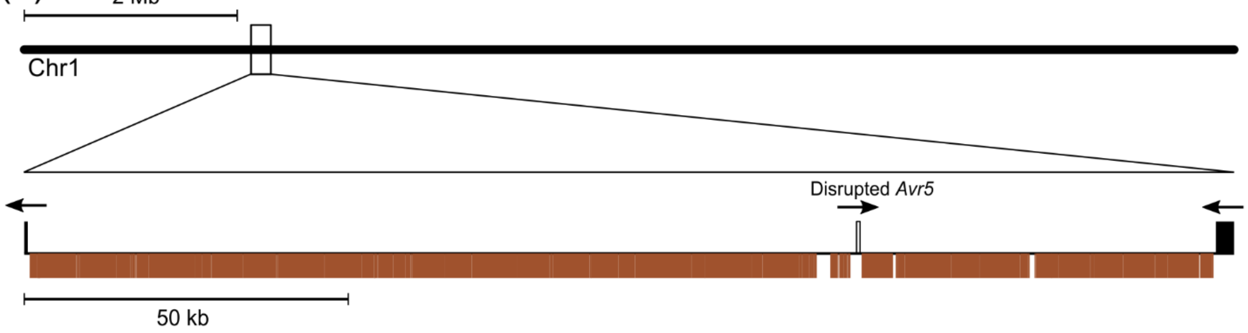

(b)

```
Chr1 : ATGAAG--TCTCCTATCGTAATCACAATTCTGGCTACTGCACTCGGGGCACTTGGTAGCTACGACGCC  >>>>     Intron 1    >>>>  CTACCTATTAACTGT 2258118
       ||||||  ||||||||||||||||||||||||||||||||||||||||||||||||||||||||||||++          51 bp          ++|||||||||||||||
Avr5 : ATGAAGTCTCTCCTATCGTAATCACAATTCTGGCTACTGCACTCGGGGCACTTGGTAGCTACGACGCCgt.........................agCTACCTATTAACTGT 81

Chr1 : AGGGACACGACCAACTACTGTTTTAACGGTAACGGACGTCACGAAGTGTG  >>>>     Intron 2    >>>>  CTCATACTGCAACCAGGCCAAAGAAGAGCCCCT 2258269
       ||||||||||||||||||||||||||||||||||||||||||||||||||++          68 bp          ++|||||||||||||||||||||||||||||||||
Avr5 : AGGGACACGACCAACTACTGTTTTAACGGTAACGGACGTCACGAAGTGTGgt.........................agCTCATACTGCAACCAGGCCAAAGAAGAGCCCCT 164

Chr1 : TAAACTAGGCCGTCGAGGAGGCCAGCGTGATTGCGGTGTAGCAGGGAGCCAATGTAACGACGTAGACCATCAGCAATGC  >>>>     Intron 3    >>>>  GATG 2258400
       |||||||||||||||||||||||||||||||||||||||||||||||||||||||||||||||||||||||||||||||++          48 bp          ++||||
Avr5 : TAAACTAGGCCGTCGAGGAGGCCAGCGTGATTGCGGTGTAGCAGGGAGCCAATGTAACGACGTAGACCATCAGCAATGCgt.........................agGATG 247

Chr1 : CCAGATGTTGCTCAAAAATAGGCTCGCCCACGTTTTATG  >>>>     Intron 4    >>>>  GAGTGCGATGCCCGTATCCGTACTAG 2258530
       |||||||||||||||||||||||||||||||||||||||++          65 bp          ++||||||||||||||||||||||||||
Avr5 : CCAGATGTTGCTCAAAAATAGGCTCGCCCACGTTTTATGgt.........................agGAGTGCGATGCCCGTATCCGTACTAG 312
```

Fig. S11. Location of *Avr5* in *Cladosporium fulvum* Race 5. (a) The disrupted avirulence gene *Avr5* is located on chromosome Chr1, next to long, repeat-rich intergenic regions. Repeats are represented as rectangles below the baseline, and genes are indicated with rectangles above the base line, with arrows indicating their transcriptional orientation. (b) Coding sequence alignment of the disrupted *Avr5* from *C. fulvum* Race 5 and *Avr5* from *C. fulvum* isolate 0WU (KJ452245.1).

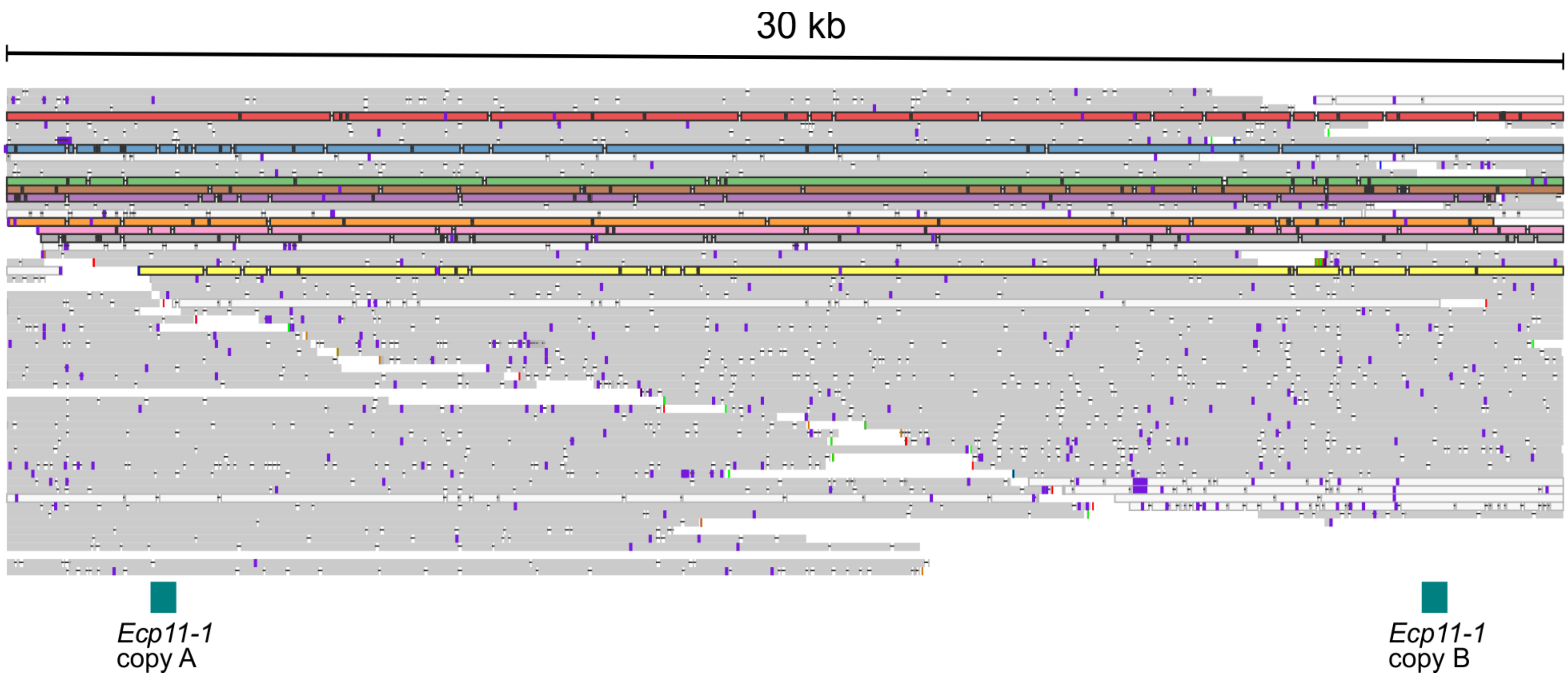

Fig. S12. The region in chromosome 5 of *Cladosporium fulvum* Race 5 containing two identical copies of the candidate effector gene *Ecp11-1*. The two gene copies (copy A and copy B) are separated by an intergenic region of 24,419 bp and both have the same transcriptional orientation. The figure shows PacBio reads mapped to the locus. Coverage ranges from 45x to 59x. The nine long reads highlighted span the entire sequences of both *Ecp11-1* copies. These nine reads were uniquely mapped to the genome of *C. fulvum* race 5 (mapping quality = 60). The figure was visualized and exported with the Integrative Genomics Viewer (IGV) v2.6.1.

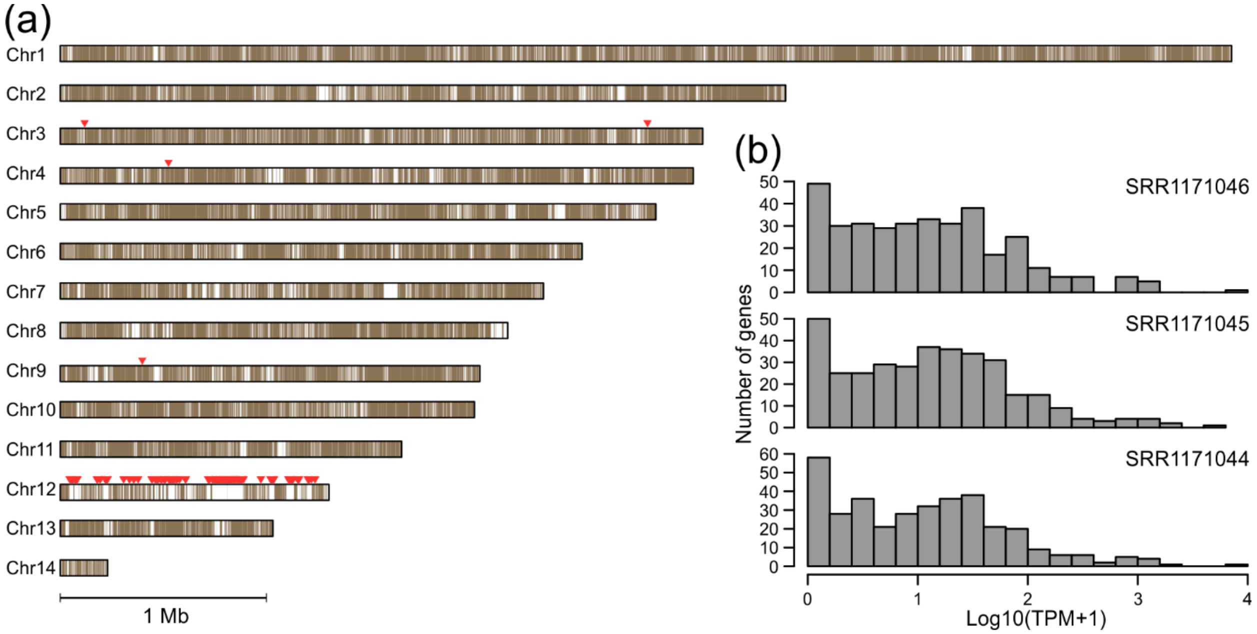

Fig. S13. Comparison of the genome assemblies of *Cladosporium fulvum* isolates Race 5 and 0WU. (a) The 14 assembled chromosomes of *C. fulvum* Race 5 are shown with filled regions indicating regions covered by *C. fulvum* 0WU contigs after mapping them with minimap2. Blank regions correspond to missing portions of the chromosomes from isolate Race 5 in the assembly of isolate 0WU. Triangles indicate 352 genes present in the assembly of *C. fulvum* Race 5 but missing in the assembly of *C. fulvum* 0WU. The figure shows that nearly half of Chr12 is not present in the assembly of *C. fulvum* 0WU and that 348 out of these 352 missing genes are in this chromosome. (b) Histogram showing the expression values in transcripts per million (TPM) of the 352 genes missing in the assembly of *C. fulvum* 0WU, based on three different RNA-seq data sets corresponding to three different conditions of isolate 0WU grown *in vitro* (SRR1171046, SRR1171045, and SRR1171044). The figure shows that most of the genes that are absent in the assembly of *C. fulvum* 0WU have clear evidence of expression based on the RNA-seq data of this isolate, indicating that these genes were misassembled in the previous reference genome of *C. fulvum* 0WU.

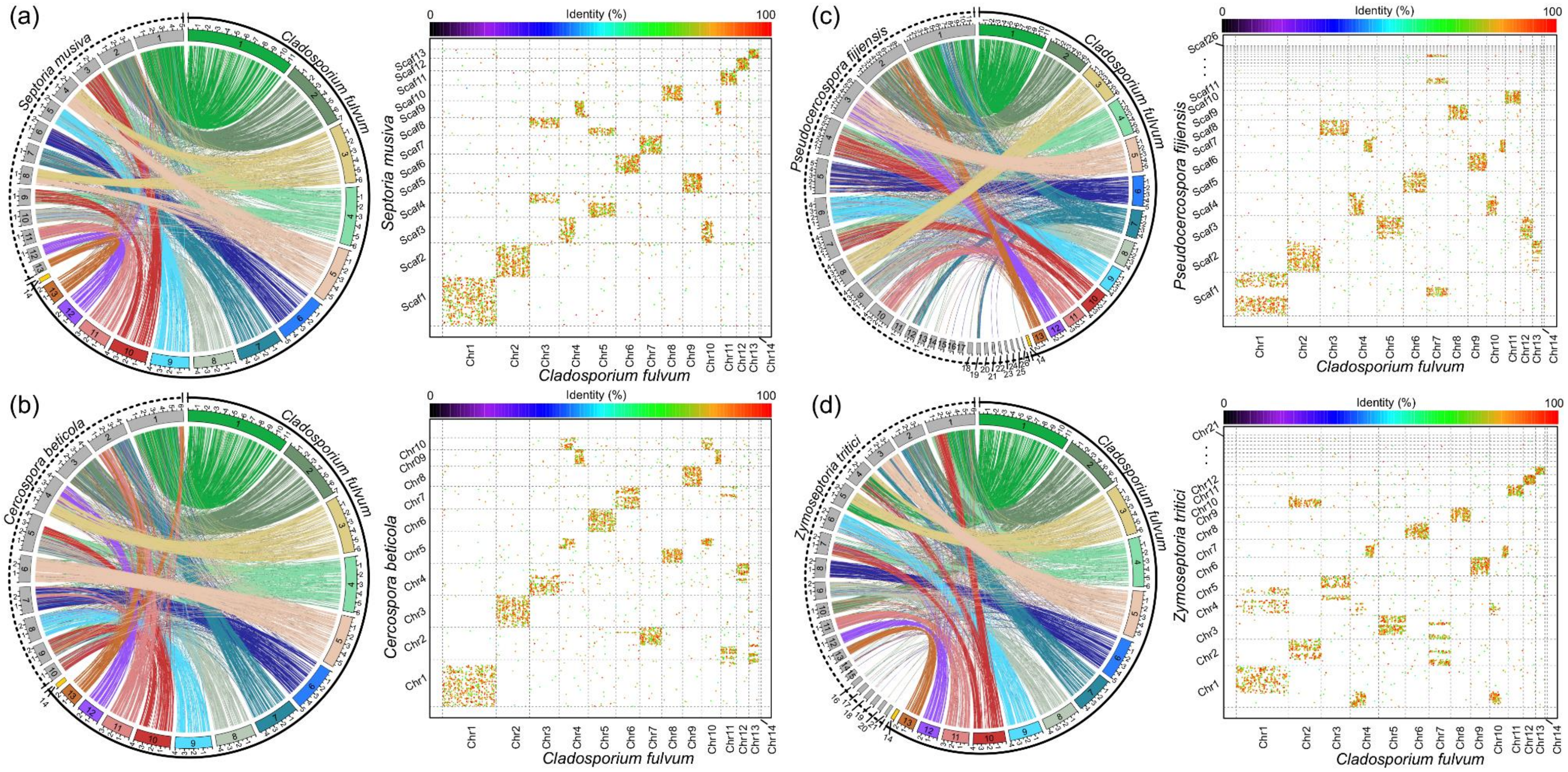

Fig. S14. Ribbon plots and dot plots showing the patterns of mesosynteny observed between *Cladosporium fulvum* Race 5 and other plant pathogenic species of Capnodiales. Genomes were aligned at the amino acid level with PROmer. Before alignment, scaffolds shorter than 100 kb were removed, and the remaining scaffolds were sorted by size in decreasing order. The 14 chromosomes of *C. fulvum* Race 5 are shown on the x-axis, and the chromosomes or scaffolds of the other species are shown on the y-axis. RefSeq accession numbers of the genomes of *Septoria musiva* isolate SO2202, *Cercospora beticola* isolate 09-40, *Pseudocercospora fijiensis* isolate CIRAD86, and *Zymoseptoria tritici* isolate IPO323 used for the alignments are GCF_000320565.1, GCF_002742065.1, GCF_000340215.1, and GCF_000219625.1, respectively.

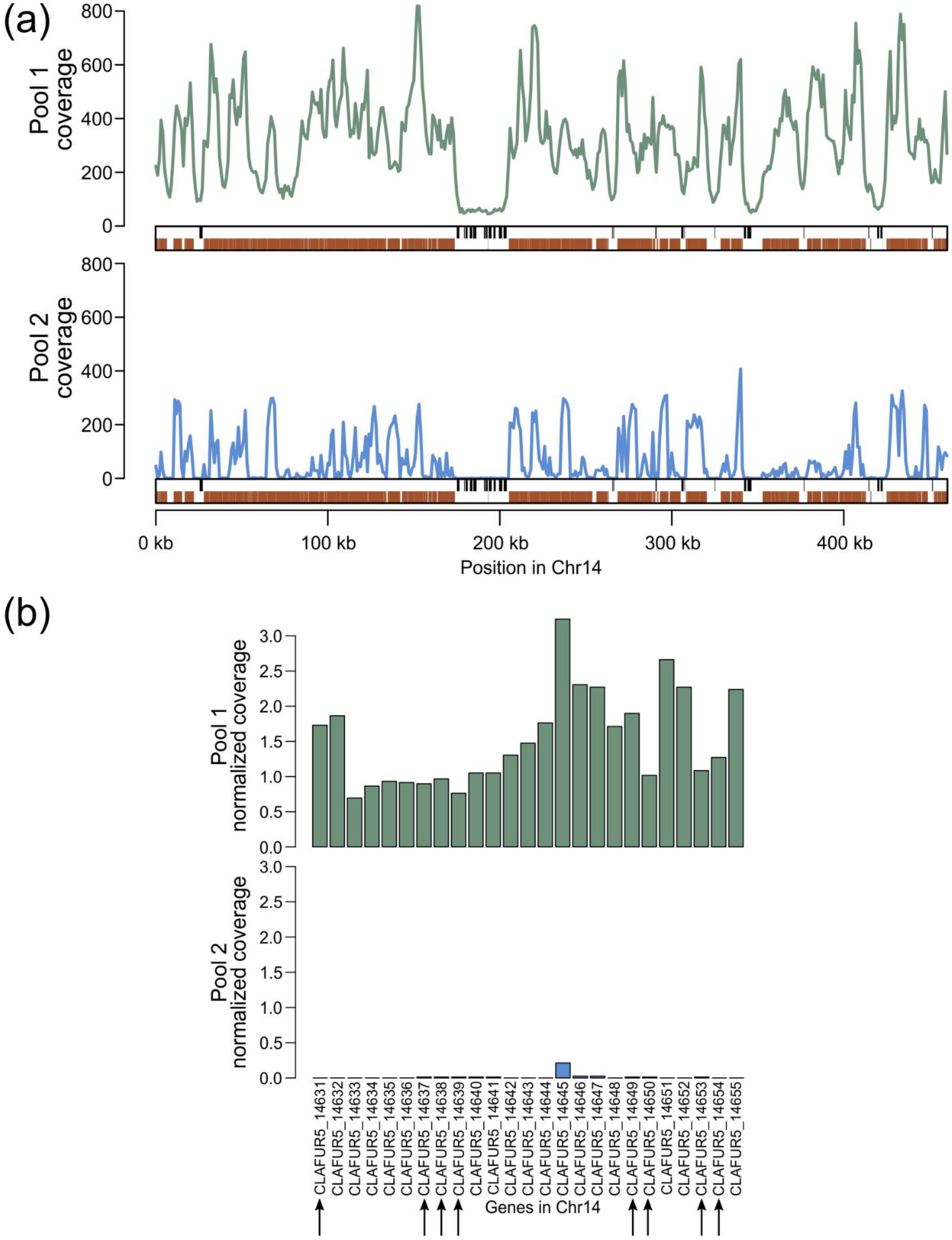
(a)
Pool 1 coverage
800
600
400
200
0
Pool 2 coverage
800
600
400
200
0
0 kb
100 kb
200 kb
300 kb
400 kb
Position in Chr14
(b)
Pool 1 normalized coverage
3.0
2.5
2.0
1.5
1.0
0.5
0.0
Pool 2 normalized coverage
3.0
2.5
2.0
1.5
1.0
0.5
0.0
CLAFUR5_14631
CLAFUR5_14632
CLAFUR5_14633
CLAFUR5_14634
CLAFUR5_14635
CLAFUR5_14636
CLAFUR5_14637
CLAFUR5_14638
CLAFUR5_14639
CLAFUR5_14640
CLAFUR5_14641
CLAFUR5_14642
CLAFUR5_14643
CLAFUR5_14644
CLAFUR5_14645
CLAFUR5_14646
CLAFUR5_14647
CLAFUR5_14648
CLAFUR5_14649
CLAFUR5_14650
CLAFUR5_14651
CLAFUR5_14652
CLAFUR5_14653
CLAFUR5_14654
CLAFUR5_14655
Genes in Chr14

Fig S15. Pooled whole-genome sequencing confirms that the mini-chromosome Chr14 of *Cladosporium fulvum* Race 5 is dispensable. *Cladosporium fulvum* isolates 2, IMI Argent 358077, and Turk 1a, for which Chr14 was predicted to be present, were grouped in pool 1, whereas isolates IPO 2.4.8.9.11 Polen, IPO 249 France, and 2.5, for which Chr14 was predicted to be absent, were pooled in pool 2. (a) Read depth across chromosome Chr14 for both pools. Predicted genes are represented with black rectangles and repetitive regions are represented with brown rectangles. (b) Bar plot showing the median coverage of all 25 genes predicted in chromosome Chr14 for pool 1 and pool 2. Coverage was normalized by the median coverage of 100 BUSCO genes (59x for pool1 and 80x for pool2). Arrows indicate PCR-amplified genes used verify the presence of Chr14 in a collection of 24 *C. fulvum* isolates (Fig S1). The figure shows that all predicted genes in Chr14 exhibits practically no coverage for the isolates in pool 2. The only exception was the gene CLAFUR5_14645, which is duplicated in *C. fulvum* Race 5 with two identical copies, one in Chr14 and the other in Chr1. In contrast, all 25 genes in Chr14 exhibited high coverage levels for isolates in pool 1.
